# Anaerobic Limonene Metabolism in a Methanogenic Enrichment Involves a Glycine Radical Enzyme

**DOI:** 10.1111/1462-2920.70192

**Published:** 2025-11-03

**Authors:** Almud Lonsing, Gerrit Alexander Martens, Anastasia Resteu, Jana Kizina, Isabella Wilkie, Alexandra Bahr, Jens Harder

**Affiliations:** ^1^ Max‐Planck Institute for Marine Microbiology Bremen Germany; ^2^ Department of Pharmaceutical Biotechnology, Institute for Pharmacy University of Greifswald Greifswald Germany

**Keywords:** Anaerolinea, hydrocarbon degradation, isoprenoids, methanogenesis, monoterpene, Velamenicoccus, wastewater

## Abstract

Limonene is a natural monoterpene omnipresent in human environments. It enters wastewater and is also metabolised in methanogenic digesters. A stable limonene‐degrading methanogenic enrichment culture was investigated by metagenomic, metatranscriptomic and metaproteomic data sets to characterise the microbial community and identify the limonene degradation pathway. Thirty‐two metagenome‐assembled genomes revealed a complex community of bacteria and methanogenic archaea dominated by *Candidatus* Velamenicoccus archaeovorus as the top predator, contributing two‐thirds of the reads in the metagenome. The presence of several fermenting bacteria (*Anaerolineaceae*, *Aminidesulfovibrio*, *Smithellaceae*, *Lentimicrobium*) indicated the recycling of necromass in a microbial loop. Only one hydrocarbon‐activating enzyme system was expressed, a member of the alkyl‐ and arylsuccinate synthase family which is a glycine radical enzyme that adds fumarate to hydrocarbons. The limonenylsuccinate synthase gene encodes a modified substrate binding pocket with two smaller amino acids, suggesting an adaptation for the larger structure of limonene. The limonenylsuccinate synthase operon and a ring cleavage operon, as well as genes for the final syntrophic fermentation to acetate, hydrogen and formate were encoded in a *Syntrophobacteraceae* genome. Almost all genes for this degradation pathway were highly transcribed and expressed, demonstrating a catalytic role for glycine radical enzymes in methanogenic systems degrading limonene.

## Graphical abstract

A methanogenic enrichment culture on the natural monoterpene limonene is characterised by a novel glycine radical enzyme for anaerobic hydrocarbon activation, the presence of a microbial loop for necromass utilisation and the presence of a predator of *B* *acteria* and *A* *rchaea*.

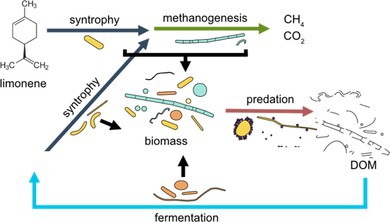

## 1 Introduction

One per mille of carbon annually fixed by plants is released into the atmosphere as monoterpenes (Messina et al. 2016). In soil, monoterpenes are released from litter and as root exudates and inhibit the oxidation of ammonium. They stimulate nitrogen fixation in the rhizosphere by serving as carbon and energy sources for heterotrophic bacteria (White 1991; Smolander et al. 2006). Monoterpenes enter the anthropogenic carbon cycle as food, for example, in orange juice and as fragrances in numerous consumer products. Although a part of it evaporates during anthropogenic processes, a substantial amount of monoterpenes reaches wastewater treatment facilities. The influent may contain up to 20 μg of limonene per litre and a broad survey in Sweden in 2004 detected 0.15–0.87 μg limonene per g dry weight of sludge (Potter et al. 2005). Clarification in wastewater treatment plants uses the biology of aerobic as well as denitrifying and methanogenic microbial communities for the transformation of monoterpenes.

Monoterpenes are isoprene‐derived hydrocarbons with carbon–carbon double bonds (alkenes). Hydrophobicity and the absence of oxygen‐containing functional groups contribute to a resistance to biodegradation. Molecular oxygen is used by many aerobic bacteria to initiate the functionalization of the alkenes (Marmulla and Harder 2014). In the absence of oxygen, the alkene may be hydroxylated in the allylic position. The nitrate‐reducing *Castellaniella defragrans* isomerizes monoterpenes (Harder and Marmulla 2017; Puentes‐Cala et al. 2018a) and then uses a limonene dehydrogenase to obtain perillyl alcohol, a functionalised compound suitable for further transformations (Puentes‐Cala et al. 2018b). Most probable number determinations have shown that for every 140 denitrifying cells in activated sludge, one is capable of growing on monoterpenes. With a population density of three million cells per millilitre, this population indicates a significant potential for reducing the concentration of monoterpenes in wastewater treatment ponds (Harder 2000). Methanogenic communities present in anaerobic digesters also consumed monoterpenes (Harder and Foss 1999). The study revealed a biological production of *p*‐cymene (*p*‐isopropylmethylbenzene) which is a major contaminant in biogas. Stable methanogenic enrichment cultures were established on limonene using initially a dilution series and maintained with one transfer of 10% v/v per year (Rotaru et al. 2012; Kizina et al. 2022). Diversity analysis based on the 16S rRNA gene showed that methane production from settled activated sludge involved a community of both *Bacteria* and *Archaea*. Additionally, the limonene‐degrading enrichment culture surprised with the abundant presence of cells affiliating to a so‐far‐uncultured phylotype OP3 originally named after the Obsidian Pool in Yellowstone, USA (Rotaru et al. 2012) and now classified as *Omnitrophota* (Seymour et al. 2023). OP3‐related cells of the phylotype LiM (for limonene‐based methanogenesis) were obtained by density centrifugation. The LiM cells‐derived genome, metaproteomes and images characterised LiM cells as predatory ultramicrobacterium that were found both free‐living and attached to other cells. It was named *Candidatus* (*Ca*.) Velamenicoccus archaeovorus (Kizina et al. 2022). As a predator, it represents a trophic level above the limonene‐degrading community of *Bacteria* and *Archaea*.

In this study, we generated metagenomes and metatranscriptomes of the whole enrichment culture to aim—in combination with existing metaproteomes (Kizina et al. 2022)—at the identification of the degradation pathway of limonene. So far, only the anaerobic degradation pathways for alkanes and aromatic compounds have been investigated in methanogenic communities. Hydrocarbon activation is catalysed by a range of anaerobic metalloenzymes.

In marine and hypersaline *Archaea*, methyl‐coenzyme M reductases (Shima et al. 2012) and alkyl‐coenzyme M reductases (Lemaire and Wagner 2022; Wegener et al. 2022; Zehnle et al. 2023) use the heterodisulfide CoM‐S‐S‐CoB as cosubstrate to oxidise a variety of alkanes to thioethers. An alternative mechanism to dehydrogenases is the addition of a carbon–hydrogen bond to fumarate by glycine radical enzymes, better known as alkyl‐ and benzylsuccinate synthases (Ass and Bss) (Heider et al. 2016; Andorfer et al. 2025). The glycine radical is introduced posttranslationally by an activase cleaving *S*‐adenosyl‐methionine into 5‐deoxyadenosine and methionine (Frey et al. 1994). The large protein A or alpha subunit of succinate synthases is widely used as a marker gene for the anaerobic activation of hydrocarbons. Methanogenesis on pentane and larger n‐alkanes is initiated by Ass present in, for example, *Smithella* within mesophilic *Syntrophaceae* (Tan et al. 2014; Ji et al. 2020) and *Peptococcaceae* species (Abu Laban et al. 2015; Siddique et al. 2020).

Aromatic compounds are also transformed by oxygen‐independent hydroxylation and carboxylation reactions, by ethylbenzene dehydrogenase (Hagel et al. 2022) and carboxylases within the superfamily of 3‐octaprenyl‐4‐hydroxybenzoate decarboxylase (UbiD family decarboxylase). Carboxylation has been established for phenol, benzene and naphthalene and was suggested for phenanthrene (Atashgahi et al. 2018; Himmelberg et al. 2018; Tomei et al. 2021; Heker et al. 2023).

This overview of the degradation of alkanes and aromatic compounds by methanogenic communities suggested several candidate enzymes for methanogenic limonene degradation. In this study, we have analysed metagenomes for the presence of these genes. Several candidate genes were identified, but metatranscriptomic and metaproteomic observations argued that limonene degradation is initiated by a limonenylsuccinate synthase in a *Syntrophobacteraceae* bacterium and results in the formation of acetate, hydrogen and formate. The metagenome also surprised with the presence of over 30 species and a preeminence of *Ca*. Velamenicoccus archaeovorus.

## 2 Materials and Methods

### 2.1 Cultivation

The methanogenic enrichment culture on limonene (1‐methyl‐4‐(1‐methylethenyl)‐cyclohex‐1‐ene) was initiated in 1997 and – as a result of a dilution‐to‐extinction isolation – 1 μL of the enrichment culture was the inoculum for the microbial diversity in culture. Since 2005, the enrichment culture has been maintained in 12 parallel lineages with one transfer per year (Harder and Foss 1999; Rotaru et al. 2012; Kizina et al. 2022). The cultures contained 300 mL freshwater methanogenic media including 2 mM acetate, 1 mM cysteine and 0.2 mM sulphide‐free FeS, 30 mL 2,2,4,6,8,8‐heptamethylnonane (HMN) and 1.5 mL of R‐(+)‐limonene in 500 mL borosilicate bottles. They were inoculated with 10% vol/vol and were incubated at 28°C with a rotary shaking of 60 rpm. Culture MM‐324 (inoculated March 15, 2014) served as inoculum for five parallel cultures that grew from 31 January 2015, till the end of October 2015. Gas formation till the end of September 2015 accumulated to 455–665 mL per culture. Since mid‐July 2015, the methane production rate was 0.29–0.43 mmol methane per L culture and day.

### 2.2 DNA and RNA Extractions and Sequencing

Biomass was obtained by differential centrifugation to separate the cells by size into three fractions. A SW28 ultracentrifuge rotor (Beckman, Palo Alto, CA) settled large and aggregated cells at 7600 rpm (7643 *g*) for 20 min (10,000 Svedberg [S]). The 10 kS cell pellet was resuspended in 1 mL 10 mM Tris, 1 mM EDTA, pH 8.0 (TE). Centrifugation was continued with the supernatant at 27,000 rpm (96,467 *g*) for 160 min (100 S). The resulting pellet was resuspended in 0.5 mL TE and centrifuged at 12,400 rpm (16,331 *g*) for 3 min (10,000 S). As a result, the pellet of 100 S aggregates contained, besides cell clumps, few single large cells, whereas the 100 S cells in the supernatant were dominated by small, free‐living cells. RNA and DNA were extracted from the three biomass fractions using the RNA PowerSoil Total RNA Isolation Kit and the RNA PowerSoil DNA Elution Accessory Kit (Qiagen, Hilden, Germany). NGS sequencing was performed by the Max Planck‐Genome‐centre Cologne, Germany (https://mpgc.mpipz.mpg.de/home/). RNA was analysed using Illumina HiSeq (150 bp reads; NCBI Bioproject PRJNA1129558, 10 kS cells: Acc. no. SRR30230645, Supporting Information Table 1.1) (Illumina, San Diego, CA). Illumina HiSeq was also applied for DNA (2 × 250 bp reads; 10 kS cells: SRR29791532, 100 S aggregates: SRR29898063 and 100 S cells: SRR29888306). Large DNA reads representing the 10 kS cells were obtained with HiFi reads on a PacBio Sequel (Pacific Biosciences, Menlo Park, CA; SRR29679608).

### 2.3 Bioinformatic Analyses

Hifi reads were assembled using the Flye de novo assembler v2.9 (Kolmogorov et al. 2020). The contigs were error‐corrected using Inspector (Chen et al. 2021), resulting in 1041 contigs with an N50 of 597,098 bp. Binning of these contigs used MaxBin v2.2.7 (Wu et al. 2015), Metabat2 v2.15 (Kang et al. 2015) and Vamb v3.0.2 (Nissen et al. 2021). DAS Tool v1.1.3 (Sieber et al. 2018) calculated a set of 32 optimal non‐redundant bins. Bin quality was estimated using CheckM v1.1.3 (Parks et al. 2015). The bin LiM6 assigned to *Syntrophobacteraceae* had a strain heterogeneity of 42.9, an indication of the presence of closely related strains. Bin quality was improved by mapping Illumina reads of 10 kS cells onto the contig LiM6 with 99% identity using bbmap v38.93 (https://sourceforge.net/projects/bbmap/) and reassembly of mapped reads together with LiM6 contigs that were set as ‘‐trusted‐contigs’ using the assembler SPAdes v3.15.3 (Bankevich et al. 2012). Contigs larger than 1500 bp were used as a starting point for an additional three rounds of assembly improvement. The refinement reduced contaminations and removed the strain heterogeneity in bin LiM6. Bins were taxonomically classified using GTDB‐Tk v1.7.0 (Chaumeil et al. 2019) based on the Genome Database Taxonomy. The bin representing *Ca*. Velamenicoccus archaeovorus had eight contigs and covered 98.46% of the closed genome (GenBank CP019384) as analysed by D‐GENIES (Cabanettes and Klopp 2018). We used the closed genome for read mapping. The relative abundance of each bin within the metagenomes was calculated using CoverM v0.6.1 (Woodcroft 2021). 22 bins had over 90% completeness and were deposited as metagenome‐assembled genomes (MAGs) at NCBI (Supporting Information Tables 1.1 and 1.2).

Proteins encoded in the 10 kS cell metagenome were annotated using Bakta v1.4.2 with its database v3.1 for bacterial genomes (Schwengers et al. 2021) and Prokka v1.14.6 for the methanogenic archaeal MAGs (Seemann 2014). Genes of interest were also annotated using the NCBI Conserved Domain Database (CDD) (Marchler‐Bauer et al. 2011). Genome annotations of *Syntrophobacteraceae* LiM6 and *Syntrophobacter fumaroxidans* (Genbank GCA_000014965.1) were analysed with DIAMOND in sensitive mode (Buchfink et al. 2021).

Metatranscriptomic analyses started with SortmeRNA v4.3.4 (Kopylova et al. 2012) to remove rRNA encoding reads. The non‐rRNA reads were trimmed with quality 30 using bbduk within bbmap v38.93 and then mapped with bowtie2 v2.4.4 (Langmead and Salzberg 2012) onto the genes of proteins present in the 10 kS cell metagenome. Transcripts per million (TPM) were calculated in R using the total mapped reads to a gene.

The analyses to detect candidate enzymes for the limonene degradation were conducted using DIAMOND v2.0.15‐BLASTP analyses (Buchfink et al. 2021) for limonene hydroxylase (NCBI acc. no. AAC25032.1), limonene dehydrogenase subunit A (CDM25290.1), limonene‐1,2‐epoxide hydrolase (CAA77012.1), limonene 1,2‐monooxygenase (CAC20855.1), methyl‐coenzyme M reductase type I (WP_013296337.1, WP_013296341.1, WP_013296338.1), methanogenic methyl‐coenzyme M reductase (WP_084174107.1, WP_042686194.1, WP_042686201.1), methyl‐coenzyme M reductase Type II (WP_013296302.1, WP_013296305.1, WP_013296303.1), alkyl‐coenzyme M reductases (RZB32666.1), phenylphosphate carboxylase (WP_245880909.1), benzene carboxylase (ADJ94002.1) and ethylbenzene hydroxylase (WP_011237152.1). Enzymes with an *E*‐value of below E‐40 and a bitscore above 100 were considered similar. These enzymes were examined in more detail with respect to their TPM, presence in the metaproteome and their annotations were additionally checked against the CDD (Marchler‐Bauer et al. 2011) and in NCBI Blast and ExPASy searches (Artimo et al. 2012; Sayers et al. 2022). To phylogenetically place the highly expressed succinate synthase alpha subunit in relation to characterised Ass and arylsuccinate synthases, amino acid sequences were aligned using the ClustalW algorithm within the MEGA11 software (version 11.0.13) with a gap opening penalty of 10 and a gap extension penalty of 0.1 (Thompson et al. 1994; Tamura et al. 2021). The alignment was used to calculate a maximum‐likelihood tree with a bootstrap value of 1000 using FastTree (version 2.1.11) (Price et al. 2009). The amino acid sequences of the proteins were retrieved from GenBank (Selesi et al. 2010; Wöhlbrand et al. 2013; Strijkstra et al. 2014; Heider et al. 2016). Alphafold 3.0.1ccd (Abramson et al. 2024) was used with default settings and SMILES line notation for limonene and fumarate. The highest confidence structure with a ranking score of 0.9635 was studied in ChimeraX (Meng et al. 2023).

For the metaproteomic analysis, the proteins of 10 kS cells, 100 S aggregates and 100 S cells were extracted and analysed by MS/MS in an LTQ Orbitrap Velos mass spectrometer (Thermo Fisher Scientific, Waltham, MA) (Kizina et al. 2022). The mass spectrometry proteomic data are available from EBI (https://www.ebi.ac.uk/pride/archive/projects/PXD025008). They were previously only used for the analysis of the *Ca*. Velamenicoccus archaeovorus proteome. Now, the quality of the PacBio metagenome enabled the use of these metaproteomes for the other community members of the limonene enrichment culture. All proteins encoded in the 10 kS cell PacBio metagenome and a set of common laboratory contaminations served as a target‐decoy database for the mass spectrometry data analysis with MaxQuant 1.6.16 (Sinitcyn et al. 2018), applying a false discovery rate on protein and peptide level of 0.01%. Protein quantity was determined in relative intensity‐based absolute quantification (riBAQ) values (Krey et al. 2014).

## 3 Results

### 3.1 Cell Fractionation and Omic Datasets

**FIGURE 1:**
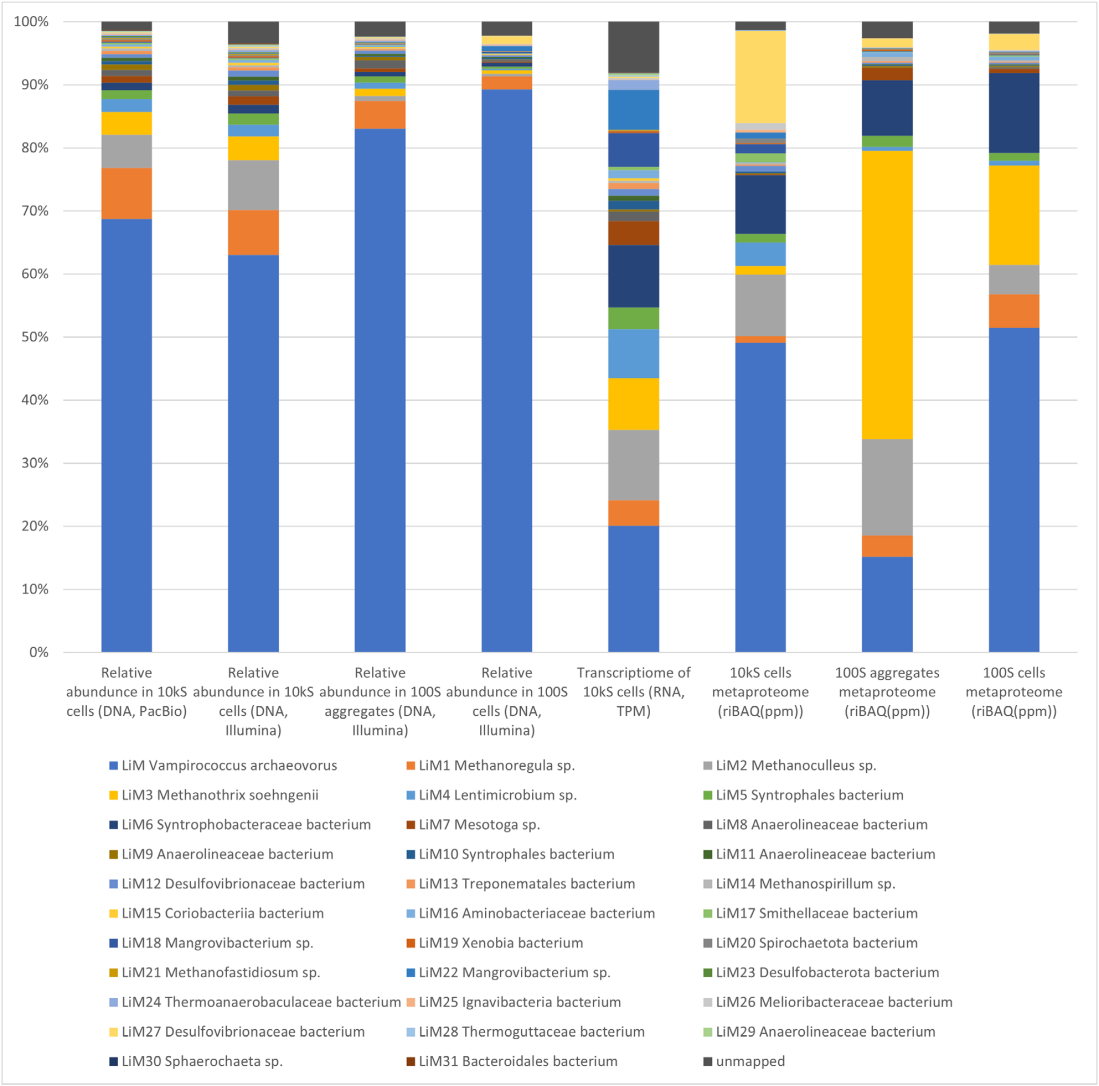
Relative abundance of metagenome‐assembled genomes in metagenome, metatranscriptome and metaproteome data.

The methanogenic community was harvested in fractions of different cell sizes. Most cells and cell aggregates were collected in the 10,000 S pellet (10 kS cells); very small cells were collected in 100 S aggregate and 100 S cell fractions (Kizina et al. 2022). Analysis of 10 kS cells by a Flye assembly of PacBio Hifi reads resulted in 32 MAGs representing a coverage of 98.73% of all reads (Figure 1, Supporting Information Table 1.3). Four MAGs were closed genomes and 22 MAGs with over 90% completeness were deposited at NCBI (Supporting Information Tables 1.1 and 1.2). The 32 MAGs were also representative of Illumina‐based metagenomes of 100 S aggregates and 100 S cells, covering at least 97.74% of all reads (Figure 1, Supporting Information Table 1.3). For the activity of the community, metatranscriptomes and metaproteomes were analysed using the contig proteins as reference (Supporting Information Table 1.4). In the large 10 kS cell metatranscriptome, 822 of 125,615 coding sequences (cds) present in the Flye assembly had a TPM value of ≥ 50 and 11,774 cds had a TPM value of ≥ 5. The metaproteome analysis detected 5696 proteins.

### 3.2 Community Members of the Limonene‐Degrading Culture

The PacBio metagenome contained 55 individual 16S rRNA gene sequences, of which 50 were assigned to 31 MAGs. Only the incomplete MAG LiM31, a *Bacteroidales*, had no 16S rRNA gene. The unassigned 16S rRNA genes affiliated with *Synergistota* (three sequences), *Sphaerochaetaceae* and *Methanobacteriota*.

### 3.3 *Ca.* Velamenicoccus archaeovorus

*Ca*. Velamenicoccus archaeovorus has been described as an ultramicrobial predator (Kizina et al. 2022). This study confirmed the genome sequence and provided quantitative insights into its abundance in the culture. The novel metagenomes revealed that the predator was the member of the microbial community with the highest abundance in the metagenome, with 68.7% of the PacBio reads mapping onto the genome (Figure 1, Supporting Information Table 1.3). For the same 10 kS cell fraction, 63% of the Illumina reads mapped to the predator genome. Coverages of 83% of the reads in the 100 S‐aggregates metagenome and 89% of reads in the 100 S cell metagenome agreed with the previously observed depletion of large cells and cell aggregates in 100 S fractions according to TEM graphs (Kizina 2017). The relative abundance of *Ca*. Velamenicoccus archaeovorus in the metatranscriptome was lower, with a contribution of 20.0% of the RNA reads of 10 kS cells (Figure 1, Supporting Information Tables 1.5 and 2). However, the intron‐containing 23S rRNA gene had not been removed by the digital filtering of rRNA reads using SortMeRNA and accounted for one‐third of the RNA reads. The metaproteome identified 1282 proteins, representing 48.1% of the protein quantity (riBAQ) in 10 kS cells. These observations suggested a top predator position for *Ca*. Velamenicoccus archaeovorus in the enclosed community.

### 3.4 Methanogenic *Archaea*

Five MAGs were identified as archaeal *Methanobacteriota*. Besides a *Methanothrix soehngenii* MAG (LiM3), two MAGs matched species according to GTDB taxonomy: *Methanoculleus* sp001896715 (LiM2) and *Methanospirillum* sp012729995 (LiM14). Novel species candidates were *Methanoregula* LiM1 and *Methanofastidius* LiM21. The latter presented below 0.5% of the omic quantities (Figure 1, Supporting Information Tables 1.3, 1.5 and 2). *M. soehngenii* LiM3 proteins revealed methanogenic activity on acetate. Eight hundred fifty‐nine proteins were detected, representing 1.3% riBAQ in 10 kS cells, along with 3.7% of DNA reads and 8.1% of RNA reads (Figure 1, Supporting Information Table 2). *Methanoculleus* LiM2 presented 9.6% of detected proteins (362 proteins) in the metaproteome, whereas *Methanoregula* LiM1 held 1.0% riBAQ (310 proteins). This suggested that *Methanoculleus* may perform the majority of hydrogenotrophic and formate‐based methanogenesis, whereas filamentous *Methanothrix* acted as the acetoclastic methanogen.

### 3.5 Bacteria

The phylum *Desulfobacterota* was represented by seven MAGs. Based on protein quantity, *Aminidesulfovibrio aminophilus* LiM27 was the second most abundant bacterium, with 14.3% riBAQ originating from 178 proteins. A second *Aminidesulfovibrio* sp., LiM12, held 0.9% riBAQ with 101 proteins. *Syntrophales* were represented by several MAGs. LiM6 was affiliated as a novel genus to *Syntrophobacteraceae*. It comprised 9.2% riBAQ calculated from 559 detected proteins. Other *Syntrophales* were *Smithellaceae* gen. nov. LiM17 and LiM5 and LiM10, which were affiliated with two novel families. LiM5 and LiM17 showed significant biomass contributions with 1.3% and 1.4% riBAQ, respectively. *Bacteroidota* accounted for 7.5% of the total riBAQ. *Lentimicrobium* LiM4 contributed 3.7% riBAQ and *Mangrovibacterium* LiM18 and LiM22 accounted for 2.3% riBAQ. *Meliobacteraceae* LiM26 of the order *Ignavibacteria* held 1.1% riBAQ. Individual MAGs of other phyla contributed little to the metaproteome. *Anaerolineaceae* was present with four species, LiM 8, 9, 11 and 29. *Spirochaetota* was present with three MAGs and a range of phyla was only represented by one MAG each: *Acidobacteriota*, *Actinobacteriota*, *Eremiobacterota*, *Planctomycetota*, *Synergistota* and *Thermotogota*. The order *Thermotogales* was represented by *Mesotoga* sp002305955 LiM7, comprising 0.082% riBAQ of the metaproteome with 123 detected proteins. Abundant *Anaerolineales* were *Anaerolineaceae* UBA4781 LiM8, comprising 33 detected proteins representing 0.053% riBAQ and *Anaerolineaceae* sp002436085 LiM11, showing 34 detected proteins accounting for 0.020% riBAQ. Altogether, the *Mesotoga* sp002305955 LiM7 and *Anaerolineaceae* MAGs comprised relatively low abundances in the metatranscriptome as well as the metaproteome, suggesting a small role in the microbial community (Figure 1, Supporting Information Tables 1.3, 1.5 and 2).

## 4 Evidence for Hydrocarbon Metabolism in the Omic Datasets

Candidate genes for hydrocarbon‐activating enzymes were identified using characterised enzymes as references in protein similarity searches with DIAMOND in BLASTp mode. The superfamily that includes benzene and phenol carboxylases was present, with three genes for quinone biosynthesis annotated as 3‐octaprenyl‐4‐hydroxybenzoate decarboxylase in the MAGs LiM13, LiM6 and LiM20. The expression level of these enzymes was below 10 TPM and the proteins were not detected in the metaproteome. The same observation was made for molybdopterin‐binding proteins of unknown function that had the highest, but overall low similarity to the ethylbenzene hydroxylase. One protein related to limonene hydroxylase was detected in the metaproteome but annotated as a transcriptional regulator. Three genes were related to limonene dehydrogenase, but they were not transcribed and expressed. Genes for the aerobic oxidation with molecular oxygen, limonene‐1,2‐monooxygenase and limonene‐1,2‐epoxide hydrolase were not present in the metagenome.

Alkyl‐coenzyme M dehydrogenase‐related proteins were annotated as six methyl‐coenzyme M reductases present in *M. soehngenii* LiM 3, *Methanofastidiosum* LiM21, *Methanospirillum* LiM14, *Methanoregula* LiM1 and twice in *Methanoculleus* LiM2. All large MCR alpha subunits were detected in the metaproteomes.

Coding sequences related to the large subunit of Bss and other glycyl radical enzymes were also frequently detected (Supporting Information Table 1.6). However, annotation varied from pyruvate formate lyase superfamily to Bss. Only one gene variant of the succinate synthase was highly transcribed. With a value of 174 TPM, it was the 261st most frequently expressed gene. In the metaproteome, it was in position 1402 of the most abundant protein in the 10 kS fraction, suggesting an increased turnover of the oxygen‐sensitive enzyme. The succinate synthase alpha subunit gene was located on a contig assigned to MAG LiM 6 belonging to the family *Syntrophobacteraceae*. This finding, together with the absence of alternative candidate enzymes for limonene activation, initiated a detailed genome analysis of MAG LiM 6 to identify the limonene degradation pathway and metabolism in this bacterium.

### 4.1 Limonene Degradation in *Syntrophobacteraceae* LiM6

**FIGURE 2:**
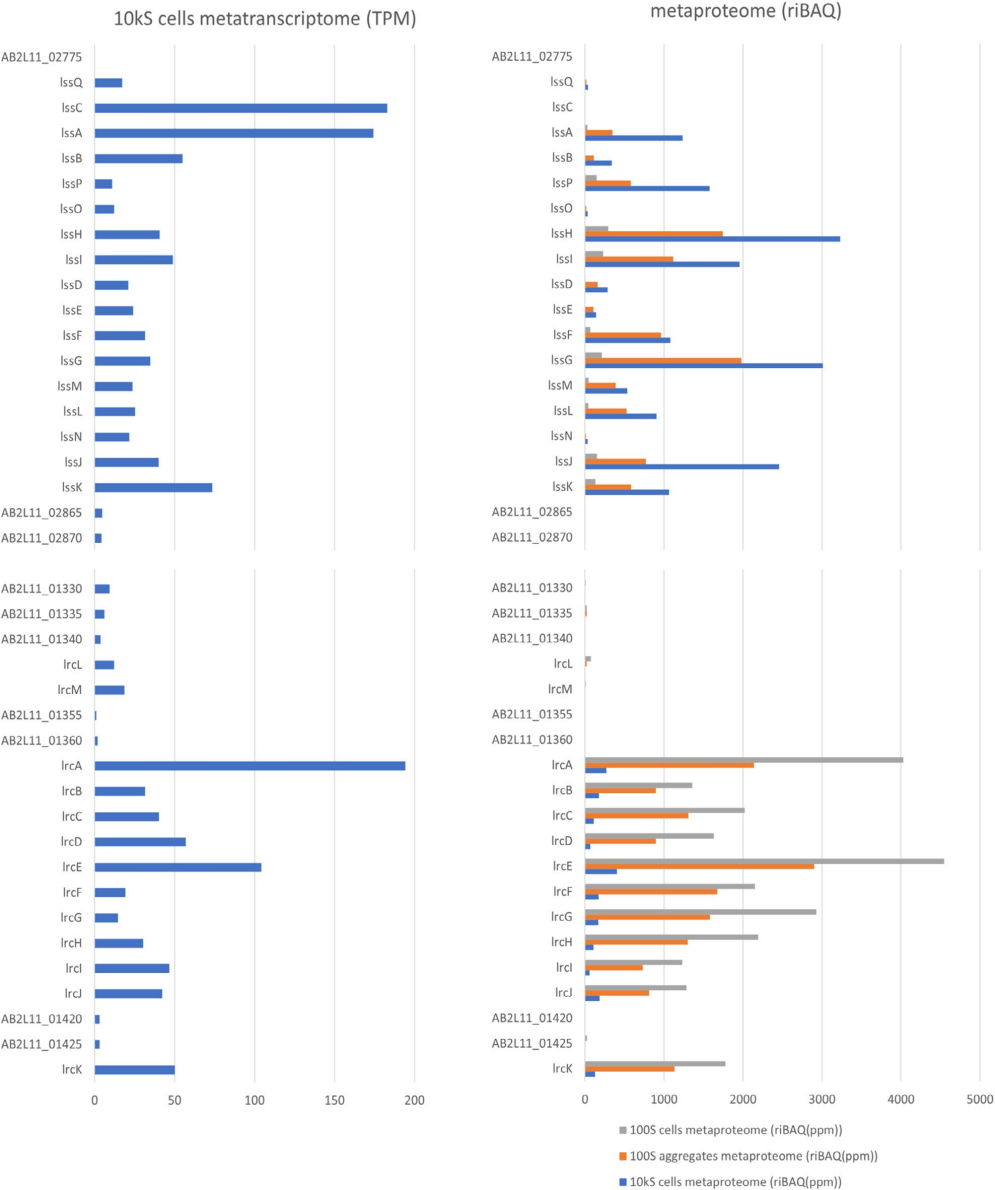
Metatranscriptomic detection of RNA and metaproteomic detection of proteins encoded in the limonenylsuccinate synthase and limonene ring cleavage operons.

**FIGURE 3:**
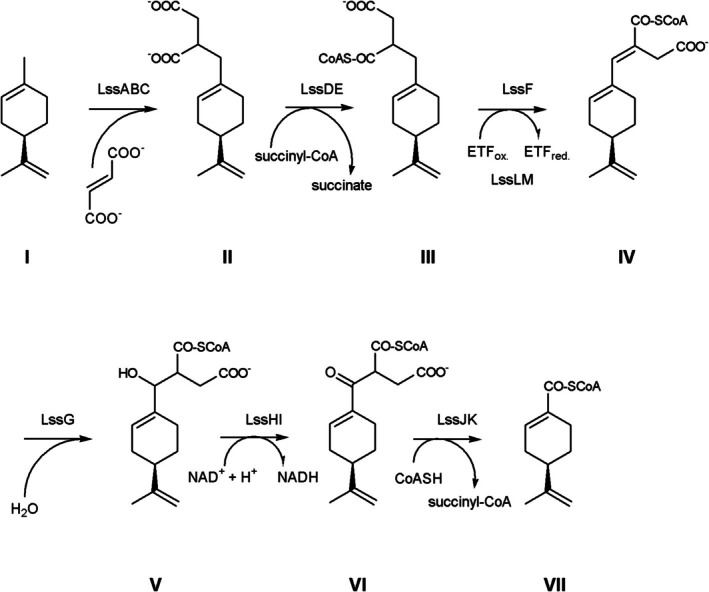
Limonene transformation to perillyl‐CoA. (I) Limonene, (II) limonen‐7‐ylsuccinate, (III) 2‐(limonen‐7‐yl)‐succinyl‐CoA, (IV) 2‐(limonen‐7‐ylidene)‐succinyl‐CoA, (V) 2‐(7‐hydroxylimonen‐7‐yl)‐succinyl‐CoA, (VI) 2‐(7‐oxolimonen‐7‐yl)‐succinyl‐CoA and (VII) perillyl‐CoA.

Hydrocarbon activation by glycyl radical enzymes needs accessory enzymes to introduce the glycine radical by *S*‐adenosylmethionine (SAM) cleavage. The genome of LiM6 encodes an expressed chaperon BssE for the activation of a glycyl radical enzyme on one contig, followed by a limonene ring cleavage *(lrc)* operon *lrcLMABCDEFGHIJK* (Figure 2). Another contig encoded the limonenylsuccinate synthase (*lss*) operon *lssQCABPOHIDEFGMLNJK*. The large abundance of these genes as transcripts in the metatranscriptome and as proteins in the metaproteome suggested the following pathway (Figure 3, manual annotation of genes in Supporting Information Table S1.7): limonene degradation is initiated by limonen‐7‐ylsuccinate synthase (*p*‐mentha‐1,8‐dien‐7‐yl‐succinate synthase) LssABC. The glycine radical enzyme adds limonene to fumarate. In the next step, the succinyl‐CoA:limonen‐7‐ylsuccinate CoA‐transferase LssDE activates a carboxylic acid and the substrate molecule is enlarged by the coenzyme A moiety providing a handle for highly specific substrate binding. A first beta‐oxidation follows, including dehydrogenation, hydration, oxidation and thiolytic cleavage. The limonene‐7‐yl‐succinyl‐CoA dehydrogenase LssF introduces the double bond in the alpha‐beta position. Electron transfer flavoproteins (ETFs) LssLM are likely the physiological electron acceptors. The limonene‐7‐ylidene‐succinyl‐CoA hydratase LssG adds water to the enoyl‐CoA ester. The alcohol is oxidised by the NAD^+^‐reducing 2‐(7‐hydroxylimonen‐7‐yl)‐succinyl‐CoA dehydrogenase LssHI. The last reaction in the beta oxidation is a thiolytic cleavage of the beta‐oxo‐acylCoA ester by 2‐(7‐oxolimonen‐7‐yl)‐succinyl‐CoA thiolase LssJK. One product is succinyl‐CoA, which is reused by LssDE. The second product is perillyl‐CoA, which is the educt for the enzymes encoded in the limonene ring cleavage (*lrc*) operon. The *lss* operon encodes also some proteins with a presumably accessory function. The ETF‐associated cytochrome b and iron–sulphur cluster‐binding oxidoreductase LssN may be involved in electron transfers, eventually bifurcation. The acyl‐CoA synthetase LssO may replenish intermediate pools of CoA esters. Two expressed proteins lack a clear function in this initial limonene degradation pathway: LssP is a protein with putative pyridoxamine 5′‐phosphate oxidase function and LssQ is a putative LolA‐like molecular chaperone for lipoproteins or other hydrophobic compounds. Of these proteins, only the small gamma subunit LssC was not detected in the metaproteome, but a value of 183 TPM in the metatranscriptome indicated relevance for the enzyme. Although located on another contig, the expression of the membrane surface proteins HflC and HflK suggested that the activation reaction may be anchored as a multiprotein complex on the membrane.

**FIGURE 4:**
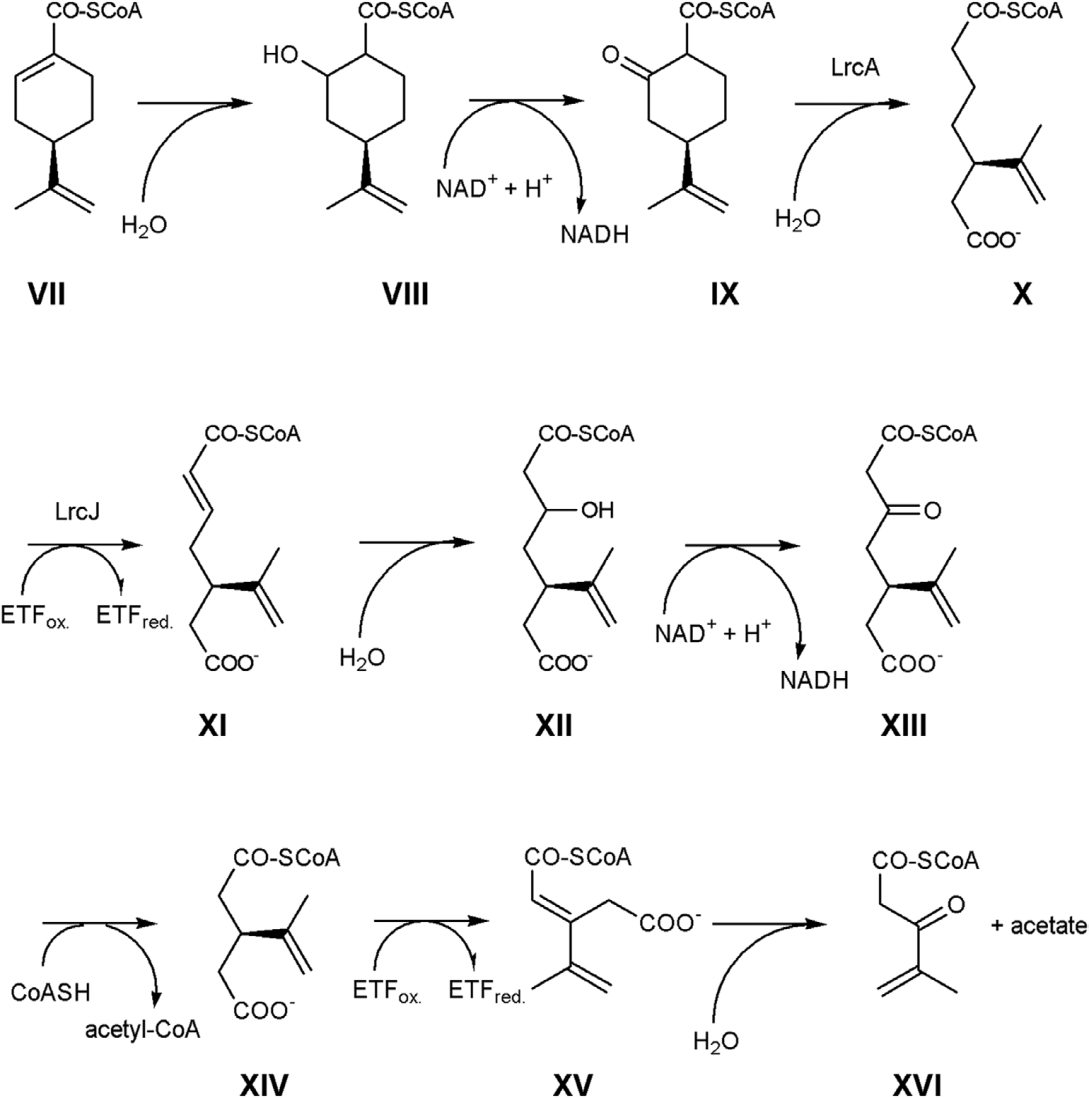
Perillyl‐CoA metabolism. (VII) Perillyl‐CoA, (VIII) 4‐isoprenyl‐2‐hydroxy‐cyclohexane‐1‐carbonyl‐CoA, (IX) 4‐isopropenyl‐2‐oxo‐cyclohexane‐1‐carbonyl‐CoA, (X): 5‐isopropenyl‐pimeloyl‐CoA, (XI) 6‐carboxy‐5‐isopropenyl‐hex‐2‐enoyl‐CoA, (XII) 3‐hydroxy‐5‐isopropenyl‐pimeloyl‐CoA, (XIII) 3‐oxo‐5‐isopropenyl‐pimeloyl‐CoA, (XIV) 3‐isopropenyl‐glutaryl‐CoA, (XV) 3‐isopropenyl‐glutaconyl‐CoA, (XVI) 4‐methyl‐3‐oxo‐pent‐4‐enoyl‐CoA.

The limonene ring cleavage (*lrc*) operon encodes BssE and enzymes for the cyclohexene ring opening and beta oxidations (Figure 4). Perillyl‐CoA hydration may occur by one of the enoyl‐CoA hydratases, LrcB or LrcF. Physiological electron acceptors may be a second pair of ETFs, EtfAB, encoded outside of the *lrc* operon. The oxidation of the alcohol 4‐isoprenyl‐2‐hydroxy‐cyclohexane‐1‐carbonyl‐CoA may be catalysed by one of the dehydrogenases: LrcE, LrcG or LrcK. The 4‐isopropenyl‐2‐oxo‐cyclohexane‐1‐carbonyl‐CoA hydrolase LrcA catalyses the ring opening to 5‐isopropenyl‐pimeloyl‐CoA. A beta oxidation with an acyl‐CoA dehydrogenase, LrcJ, an enoyl‐CoA hydratase (LrcB or LrcF), a dehydrogenase (LrcE, LrcG or LrcK) and a thiolase (LrcD or LrcH) results in 3‐isopropenyl‐glutaryl‐CoA. 3‐modified glutaryl‐CoA can be functionalised by acyl‐CoA dehydrogenase and enoyl‐CoA hydratase to a tertiary alcohol; in this case, 3‐hydroxy‐3‐isopropenyl‐glutaryl‐CoA. LrcC, an enzyme of the 3‐hydroxy‐3‐methyl‐glutaryl‐CoA synthase superfamily, cleaves at the tertiary carbon of 3‐hydroxy‐3‐isopropenyl‐glutaryl‐CoA into acetyl‐CoA and 4‐methyl‐3‐oxo‐pent‐4‐enoyl‐CoA, the end product of this operon. Expressed proteins also include an acyl‐CoA:acetate CoA transferase, LrcI and two proteins with domains of unknown functions, LrcL (DUF4125) and LrcM (DUF4037). Adjacent operons include an expressed beta‐ketoacyl‐acyl carrier protein reductase and a tripartite transmembrane efflux transporter, MepABC (monoterpene‐associated efflux proteins).

The degradation of 4‐methyl‐3‐oxo‐pent‐4‐enoyl‐CoA may start with a thiolytic cleavage into 2‐methyl‐propenoyl‐CoA and acetyl‐CoA. Hydration and two oxidation reactions yield methylmalonyl‐CoA. Carbon‐skeleton rearrangement yields succinyl‐CoA, which can be transformed into fumarate and further to oxaloacetate. After decarboxylation, pyruvate can be oxidised to acetate. The proteome provided strong support for this pathway by the presence of the methylmalonyl‐CoA mutase small and large subunits. The importance of the B_12_‐enzyme is highlighted by the expression of a cobalt ABC transporter, a B_12_‐binding protein and a corrinoid adenosyltransferase. After hydration, an oxaloacetate‐decarboxylating malate dehydrogenase produces pyruvate. Pyruvate oxidation can be catalysed by a 2‐oxoacid:ferredoxin reductase, providing reducing equivalents at a very negative electrochemical potential for proton or carbonate reduction. Acetyl‐CoA transformation to acetate enables energy conservation by an acetyl‐CoA synthetase.

*Syntrophobacteraceae* are well known for multiple hydrogenases (Hases) and formate dehydrogenases (FDHs); that is, *S. fumaroxidans* has 8 Hases and 6 FDHs (Mollaei et al. 2021). Strain LiM expresses a locus encoding two FDH alpha subunits, one FDH beta subunit, a NiFe Hase alpha subunit, a NiFe Hase 20 kDa subunit (NADH acceptor oxidoreductase), a ferredoxin reductase and a ferredoxin. A second locus codes for expressed FDH subunits, an NADH:acceptor oxidoreductase and Hase maturation factors. HypB, a nickel incorporation factor for Hases, was also expressed. Another NiFe Hase locus is located beside the aforementioned cobalt ABC transporter. A group of redox‐active proteins was also expressed and annotations involved NAD‐reducing Hase subunit HndC. These proteins are involved in the electron transfers involving ferredoxin, NADH and ETFs and likely include bifurcation and reverse electron transport. Expressed energy conservation proteins include a pyrophosphate‐energised proton pump and ATP synthase.

The proteome included also enzymes of the three‐carbon‐compound part of glycolysis and additional acyl‐CoA dehydrogenases, enoyl‐CoA hydratases, dehydrogenases and thiolases. Enzymes with a presumably biosynthetic function were 3‐isopropyl‐malate dehydratase and acetolactate synthase.

### 4.2 Limonenylsuccinate Synthase

**FIGURE 5:**
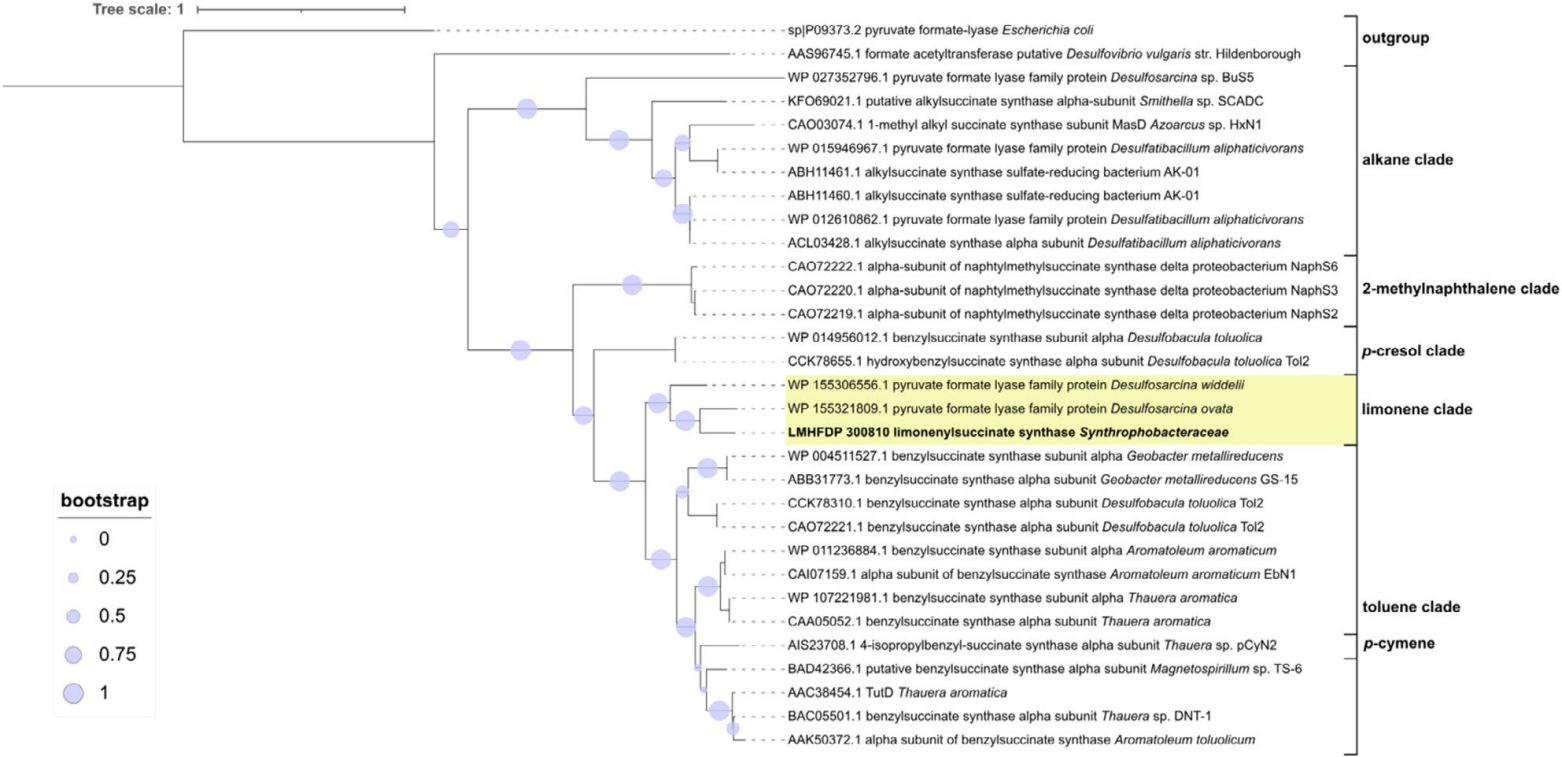
Maximum likelihood phylogenetic tree displaying the relationship of the limonenylsuccinate synthase alpha subunit with other glycine radical enzymes. The scale bar indicates the number of substitutions per site. Sequences of glycine radical enzymes were obtained from (Grundmann et al. 2008; Selesi et al. 2010; Wöhlbrand et al. 2013; Strijkstra et al. 2014; Heider et al. 2016). Supporting Information contains the corresponding alignment.

The phylogenetic analysis placed the limonenylsuccinate synthase alpha subunit together with desulfobacterial enzymes from *Desulfosarcina widdelii* and *Desulfosarcina ovata* in a new clade within the branch of naphthylmethylsuccinate synthase and Bss and separate from Ass (Figure 5). The new clade is more related to the toluene clade than to the *p*‐cresol and the 2‐methylnaphthalene clades and comprises proteins from *Deltaproteobacteria*. This is not exclusive; *Deltaproteobacteria* also encode Ass and Bss.

Bss are well characterised. The substrate binding pocket of the active site was defined by crystal structures of the enzyme with toluene and/or fumarate (Funk et al. 2015; Heider et al. 2016). For the large alpha subunit, the limonenylsuccinate synthase shares 61% identical amino acids with the Bss alpha subunit of *Thauera aromatica* (Supporting Information Data). Amino acids involved in fumarate binding and catalysis by Bss are conserved in the limonenylsuccinate synthase. Some differences are located in the substrate binding pocket of the active site (Funk et al. 2015; Heider et al. 2016). At the bottom of the substrate binding pocket, Ile‐384 (nomenclature of the Bss from *T. aromatica* ) blocks the space opposite the 4‐position of the benzyl ring. It is replaced by smaller amino acids in succinate synthases with a large substituent in the 4‐position: a threonine in limonenylsuccinate synthase and a valine in 4‐isopropylbenzyl‐succinate synthase of pCyN2. Deltaproteobacterial glycine radical proteins within the limonene clade (i.e., WP_155306556.1 from *D. widdelii* and WP_155321809.1 from *D. ovata* ) have alanine, a different amino acid at this position and may not transform limonene. Substitution of the large isoleucine Ile‐617 by valine enlarges the substrate cavity. The mutant Val‐617 (I617V) lifts a steric hindrance and enables the activation of *m*‐xylene by Bss (Salii et al. 2021). Limonenylsuccinate synthase and naphthylmethylsuccinate synthase have a histidine at this position, which is also smaller than isoleucine and in the size range of valine. The space requirement of limonene is due to its structure: unlike aromatic compounds, limonene is not a planar ring.

**FIGURE 6:**
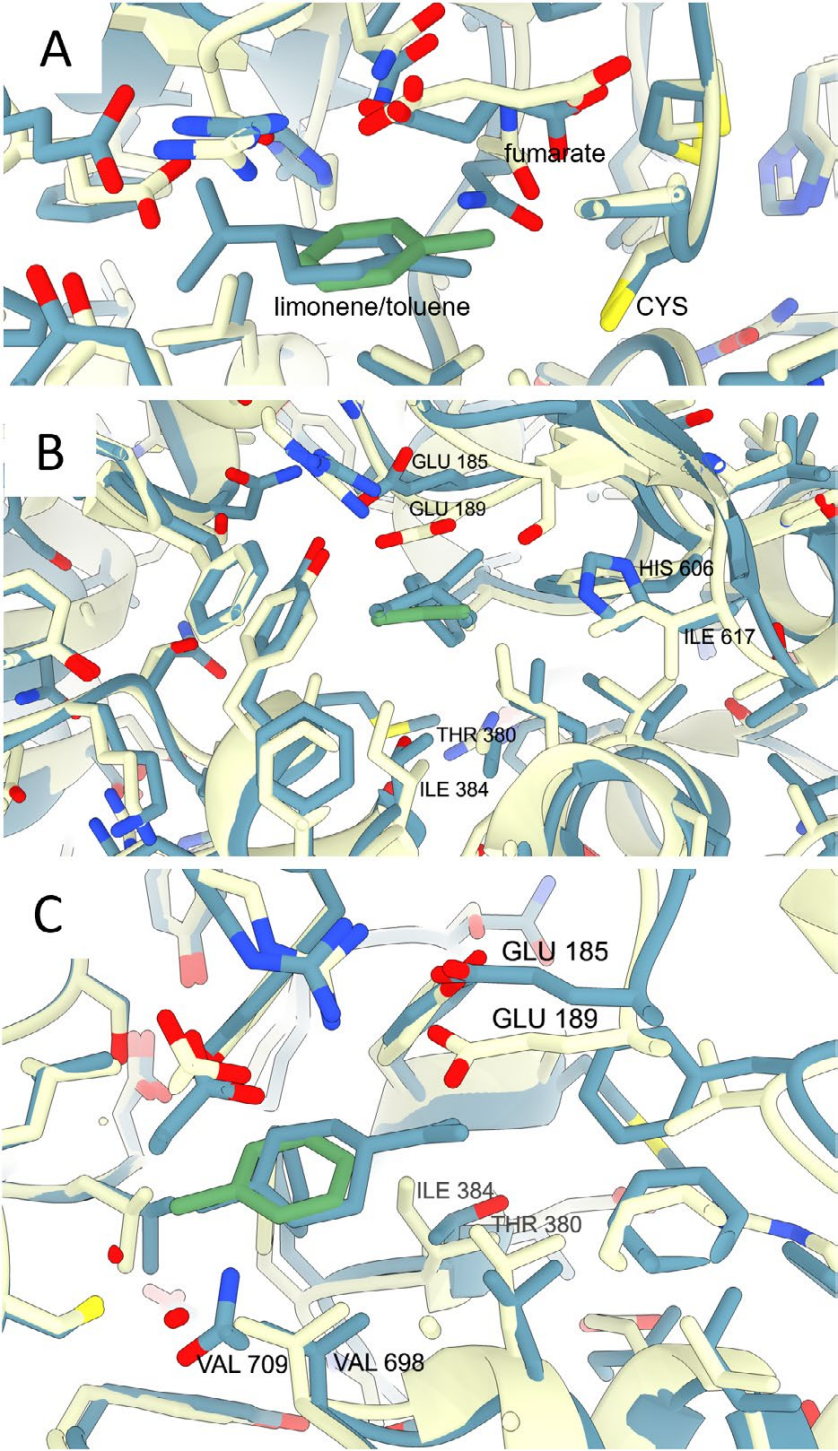
Superimposed Alphafold3 model of limonenylsuccinate synthase (blue) and crystal structure of benzylsuccinate synthase (PDB:5BWE, light yellow), both with substrate and fumarate. (A) conserved reaction side with essential cysteine, fumarate and the substrates limonene (blue) and toluene (green), (B, C) variations in the substrate binding side as viewed from the aromatic ring (B) and a side view on the isopropenyl residue of limonene. Lss Thr380 and His606 use less space than the isoleucines in Bss. Lss Glu185 is well separated from Bss Glu189. The sequence alignment score of the superimposition was 3132.4, the root mean square deviation of atomic positions was 0.811 Å for 813 pruned atom pairs and 1.004 Å across all 850 pairs.

These predictions were tested with an Alphafold3 model of the alpha subunit of limonenylsuccinate synthase with limonene and fumarate as ligands. The model was superimposed with the alpha subunit of the alpha‐beta‐gamma dimeric complex of Bss with bound toluene and fumarate (PDB:5bwe, Funk et al. 2015) (Figure 6). Fumarate addition to a methyl group requires a catalytic cysteine, Bss Cys 493. The reaction side is highly conserved, with Cys, hydrocarbon methyl group and fumarate located at nearly the same position in the superimposed structures (Figure 6A). Atomic distances of the methyl carbon to the catalytic cysteine and fumarate C2, C3 carbons are very similar. Both aforementioned amino acid differences between Lss and Bss in the active side concern the substrate binding pocket (Figure 6B,C). Both Lss His606 and Thr380 side chains provide, in comparison to Bss Ile384 and Ile617, a little more space in the substrate pocket. The isopropenyl side chain of limonene fits into the substrate pocket due to a shift of a glutamate residue (Bss Glu189 and Lss Glu185). This unexpected observation coincides with a high flexibility of the Bss secondary structures during catalysis in which an open enzyme structure closes during substrate binding. The isopropenyl side chain of limonene seems to stop the movement of the glutamate by its physical presence. This is supported by Bss Glu189 functions. It is part of the substrate channel and of the polar network on one side of the substrate binding side, involving Arg508 and fumarate. It is replaced by the smaller Asp in naphthylmethylsuccinate synthase and Gln in 4‐hydroxybenzylsuccinate synthase (Funk et al. 2015).

### 4.3 Physiology of Other Bacteria

The predatory activity of *Ca*. Velamenicoccus archaeovorus leaves membrane remains, as previously seen on TEM images (Kizina et al. 2022), and likely also cytoplasmic content for a chemoheterotrophic community. These remains were used within a microbial loop for methanogenesis. The metaproteome indicated the specific metabolic activity of individual species.

*A. aminophilus* LiM27 and *Aminidesulfovibro* sp. LiM12 expressed several amino acid ABC transporters and FDHs and hydrogenases for a syntrophic peptide fermentation. Peptidases and ABC transporters suggested a role of *Mesotoga* LiM7 in protein utilisation. Enzymes for long fatty acid degradation were expressed by *Syntrophales* (LiM5, LiM10 and *Smithellaceae* LiM17), including propionate metabolism by LiM5 (propionyl‐CoA carboxylase). Glycerol, likely released from lipids, was a major substrate for *Anaerolinaceae*, using glycerol and dihydroxyacetone kinases. Detection of an ethanolamine utilisation microcompartment protein EutM revealed the participation of a *Melioribacteriaceae* bacterium in the mineralisation of phospholipids. Surface proteins and SusCD transporter for oligosaccharides supported the hypothesised role of *Bacteroidota* for sugar degradation. *Lentimicrobium* had expressed a large number of surface proteins, but only one SusCD pair, in agreement with the narrow substrate range of *Lentimicrobium saccharophilum*. In contrast, *Mangrovibacterium* LiM18 and LiM22 had several expressed SusCD pairs. The expression of a nitrogenase by *Mangrovibacterium* LiM18 indicated a nitrogen limitation in the medium. Genes annotated as exonucleases were encoded in several MAGs, but not detected in the metaproteome.

## 5 Discussion

Our meta‐omic analysis of a methanogenic enrichment culture growing on limonene revealed an unusually broad bacterial community and a glycyl radical enzyme‐based degradation pathway for limonene.

Methanogenic communities in mesophilic hydrocarbon enrichments (Embree et al. 2015; Tan et al. 2015; Fowler et al. 2016) are characterised by a syntrophic community of *Bacteria* and *Archaea*. An abundant bacterium activates and degrades the hydrocarbon, accompanied by few other bacterial species that may provide essential amino acids or equilibrate concentrations of hydrogen and carbon dioxide to formate to the same thermodynamic energy level. The archaeal community is usually composed of the acetoclastic *Methanothrix* and *Methanoculleus*, *Methanoregula* and *Methanospirillum*, which use hydrogen and carbon dioxide or formate as substrate. *Methanothrix*—also validly published as *Methanosaeta* under the International Code of Nomenclature of Prokaryotes—is expected because the genus is known as the oligotrophic acetoclastic methanogen with the highest affinity for acetate (Jetten et al. 1992). The ecological dominance of individual hydrogenotrophic methanogenic species is less understood.

**FIGURE 7:**
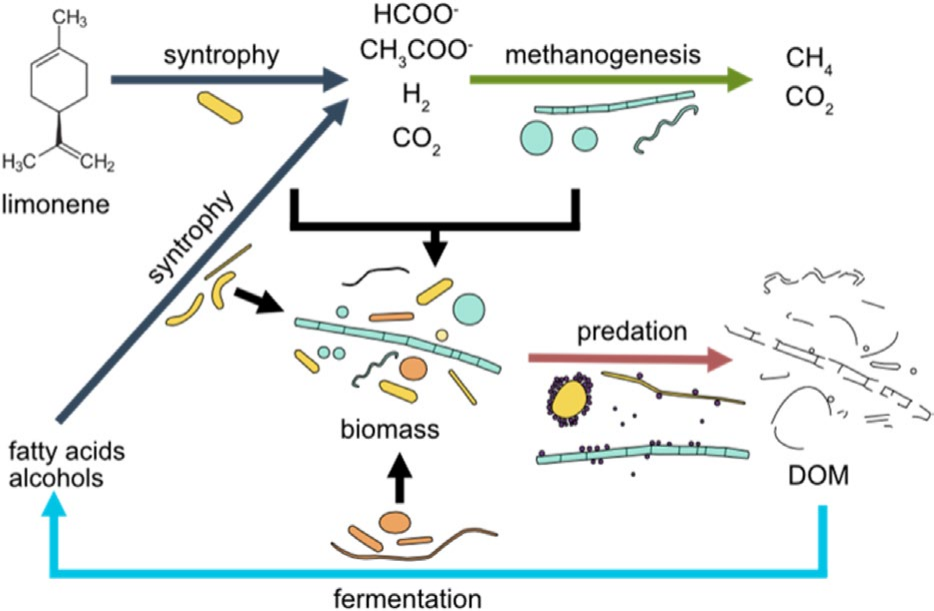
A microbial loop based on the predatory release of dissolved organic matter in the limonene‐degrading methanogenic enrichment culture. Limonene as substrate is metabolised by *Syntrophobacteraceae* LiM6 and methanogens. This trophic level is predated by *Ca*. Velamenicoccus archaeovorus. Cellular components provide dissolved organic matter (DOM) substrate for a second community of fermenters and syntrophs together with methanogens. This community is also predated, contributing to the enrichment of predatory cells.

The limonene enrichment has an archaeal community similar to other methanogenic hydrocarbon enrichment cultures. It is dominated by *Methanothrix* and *Methanoculleus*, but other species are also present. *Ca*. Methanofastidiosum has a narrow metabolism; it combines methylthiol reduction with hydrogen oxidation (Nobu et al. 2016). Its presence in the community is an indication of an active sulphur metabolism in the enrichment culture. The bacterial community was far larger than expected, with 27 metagenome‐derived genomes. The predating *Ca*. Velamenicoccus archaeovorus nourishes on the biomass of the first trophic layer presented by syntrophs and methanogens. It did not attack and digest itself, as evidenced by the huge abundance in omic datasets together with earlier electron microscopic observations (Kizina et al. 2022). The predation process likely releases prey cytosolic compounds, and cell membranes remain as observed in electron micrographs (Kizina et al. 2022). This organic matter is the substrate for a community of fermenters, synthrophs and methanogens. In aquatic systems, the utilisation of dissolved organic matter from dead biomass (necromass) by microorganisms is named the microbial loop, yielding more biomass in higher trophic levels (Azam et al. 1983). In analogy, the leftovers of the predation by *Ca*. Velamenicoccus archaeovorus are recycled by a syntrophic community, resulting in more prey cells for the predator (Figure 7). This necromass utilisation contributed to the microbial diversity in the enrichment culture. Evidence for individual microbial niches was obtained from metaproteomes. We suggest that proteins are the major substrate for *A. aminophilus*, an established amino acid‐fermenting and sulphate‐reducing bacterium initially isolated and validly described as *Desulfovibrio aminophilus* (Baena et al. 1998). Lipids include fatty acids, glycerol and ethanolamine. The metaproteome indicated that fatty acids are primarily used by *Syntrophales* LiM5, glycerol by *Anaerolinaceae* spp. and ethanolamine by *Melioribacteriaceae* bacterium LiM26. Polysaccharides are preferred substrates for *Bacteroidia*. These primary and secondary fermentations yield substrates for methanogenic *Archaea*. The presence of *Anaerolineaceae* in slowly‐growing methanogenic enrichment cultures was previously noticed and also linked to necromass utilisation (Liang et al. 2016; Liu et al. 2023). Experimental evidence for necromass utilisation in anaerobic communities was previously demonstrated by cocultivation of a naphthalene degrading sulphate reducer in combination with a necromass utilising spirochaete (Dong et al. 2018). This anaerobic microbial loop contributes to the microbial utilisation of a variety of substrates by specialised bacteria and maximises organic matter mineralisation and species diversity in the enrichment.

A key to the stable maintenance of the complex limonene‐degrading community with a microbial top predator is a slow nutrient‐limited growth rate of the first trophic level, the methanogenic community, allowing the predatory bacteria to thrive at least at equal growth rates to the organisms of the first trophic level. So far, members of the candidate phylum Omnitrophota closely related to *Ca*. Velamenicoccus archaeovorus have only been observed in slowly growing methanogenic enrichment cultures on limonene and on short chain fatty acids (Jackson et al. 1999; McInerney et al. 2008). Anaerobic predatory communities may also be experimentally established using long stationary phases to give the predator time to proliferate.

For the degradation of limonene into small molecules, metatranscriptomic and metaproteomic observations revealed the expression of two operons from the genome of *Syntrophobacteraceae* LiM6. Key enzymes are limonenylsuccinate synthase and 4‐isopropenyl‐2‐oxo‐cyclohexane‐1‐carbonyl‐CoA hydrolase, which activate limonene and catalyse the cyclohexane ring opening, respectively. The annotation was based on high amino acid similarity to well‐characterised enzymes. The limonene‐activating enzyme in denitrifying bacteria, the limonene dehydrogenase of *C. defragrans* , was not detected in the methanogenic culture. It requires an electron acceptor with a positive reduction potential (Puentes‐Cala et al. 2018b) and, hence, may not be operable in microorganisms living in a highly reduced environment, that is, sulphate‐reducing or methanogenic systems. This study has revealed a novel pathway for the degradation of limonene using a glycine radical enzyme. These enzymes are oxygen‐sensitive and used in facultative and obligate anaerobes as a radical source in many reactions involving radical intermediates. Our findings revealed an additional use of the radical enzyme, the anaerobic functionalization of the alkene limonene to limonenylsuccinate.

## Author Contributions

**Almud Lonsing:** investigation, validation, data curation, visualisation, writing – original draft, writing – review and editing. **Gerrit Alexander Martens:** investigation, writing – review and editing. **Anastasia Resteu:** investigation, writing – review and editing. **Jana Kizina:** investigation, writing – review and editing. **Isabella Wilkie:** investigation, writing – review and editing. **Alexandra Bahr:** investigation, writing – review and editing. **Jens Harder:** conceptualization, supervision, validation, visualisation, writing – original draft, writing – review and editing.

## Conflicts of Interest

The authors declare no conflicts of interest.

## Supporting information


**Data S1:** emi70192‐sup‐0001‐supinfo.pdf.

**Table S1:** Experimental datasets.

**Table S2:** Metagenome‐derived genomes in omic data sets.

## Data Availability

Proteomic data are available at PRIDE (PXD025008). NGS sequences and MAGs are deposited at NCBI.
