# Supplementary material for: Anaerobic Limonene Metabolism in a Methanogenic Enrichment Involves a Glycine Radical Enzyme
Source: Environ Microbiol. 2025 Nov 3;27(11):e70192. doi: 10.1111/1462-2920.70192 (PMC12583861; doi:10.1111/1462-2920.70192)
Supplement: Supplementary file 1 — Data S1: emi70192‐sup‐0001‐supinfo.pdf. [file EMI-27-e70192-s002.pdf]

Supplementary data

Anaerobic limonene metabolism in a methanogenic enrichment involves a glycine radical enzyme

Almud Lonsing[1], Gerrit Alexander Martens[1], Anastasia Resteu[1], Jana Kizina[1], Isabella Wilkie[1], Alexandra Bahr[2], Jens Harder[1*]

[1] Max-Planck Institute for Marine Microbiology, Bremen, Germany

[2] Department of Pharmaceutical Biotechnology, Institute for Pharmacy, University of Greifswald, Greifswald, Germany

Corresponding author: Jens Harder, jharder@mpi-bremen.de

1. Blastp Alignment Lss against Bss (5bwe)
2. Alignment of glycine radical enzymes (see Fig. 5 for tree)

1. Blastp Alignment Lss against Bss (5bwe)

```
Query: LMHFDP_300810 limonenylsuccinate synthase Synthrophobacteraceae ID:
lcl|Query_5545863(amino acid) Length: 859
Subject:5BWE_1|Chains A, D|benzylsuccinate synthase alpha chain|Thauera
aromatica (59405) ID: lcl|Query_5545865(amino acid) Length: 878


Lss     7     VEYKGKVIDFPFDNPKEIDVPPDQLAETLVQPSTERTRRLKARCRYKHTAAGEFVDPNTK  66
              +EYKGK ++F  ++P E  +P D+L E L +PST RT+RLK RCR+KH +AGEF++ +
5bwe    10    LEYKGKKLNFTPEDPAEETIPADELHEHLQKPSTARTKRLKERCRWKHASAGEFIEKSVT  69

Lss     67    GGIERMRYYTEAHRASEGEIQVLRRAKCLANFLNKMTLVIQEDELVVGSNAEHPDWIPLH  126
               GIERMRY TEAH+ASEG+ + +RRA  LAN LNK TLV+QEDE +VG +AE P+  PL+
5bwe    70    AGIERMRYLTEAHKASEGKPEAIRRALGLANVLNKSTLVLQEDEFIVGYHAEDPNMFPLY  129

Lss     127   PETSFVNVIDFVNSPYCPEEKEEAIEYAQYWARHSIMYKGERYFTREELDQQYQFSTMEA  186
              PE S + V D++ S Y P+  +EA    +YW  HS+  K + YF   +L + YQ S+MEA
5bwe    130   PELSHMAVQDYLRSDYSPQPADEAAAINEYWKPHSLQSKCQPYFDPADLGRMYQVSSMEA  189

Lss     187   PTFANAYNSTVPPYETIMEDGLLKRIAMCKENIAKAKAELAAPDWNGPERLPLCDKIDQW  246
              P+FA+ YNS VPPYET++EDGLL RI + +++IA+A+A+++   WNG + L    KID W
5bwe    190   PSFASGYNSIVPPYETVLEDGLLARIKLAEKHIAEAQADMSTFPWNGTKGLDNIAKIDNW  249
```

```
Lss     247  DAMIMADEAVIAWAQRYGRLAKIIAENFESDPKRKTELLEIADICHRTPAEPCKGLRDAM  306
              AM++A +AVI+WA+R GRL KI+AENFE+DPKR+ ELLEIADIC R PAEPCKGL+DAM
5bwe    250  KAMVIACKAVISWARRQGRLCKIVAENFETDPKRQAELLEIADICQRIPAEPCKGLKDAM  309

Lss     307  QVKWFTYLVCHSLEAYASGYAHKEDKVLWPYYKMSVIDKSFQPMTHADAVELVECERLKV  366
             Q K+FT+L+CH++E YASGYA KED +LWPYYK SV+DK FQPM+H DAVELVE ERLK+
5bwe    310  QAKFFTFLICHAIERYASGYAQKEDTLLWPYYKASVVDKKFQPMSHMDAVELVEMERLKI  369

Lss     367  SEHGCAKNRMYRITFAGSNDLFILSVGGLNSDGTDGCNDMTDAILEAARNIRTTEPSIGF  426
             SEHG  K+R YR  F GSNDLFIL+VGG N+ G D CNDMTDAILEAA+ IRT EPSI F
5bwe    370  SEHGAGKSRAYREIFPGSNDLFILTVGGTNAKGEDACNDMTDAILEAAKRIRTAEPSIVF  429

Lss     427  RWNKIGREKTKRLVFECIRDGLGYPSIKNDELNTDQL-------INAFGATPEEARDWAL  479
             R++K  REKT R VFECIRDGLGYPSIK+DE+ T+Q+       +N  GAT EEA +W
5bwe    430  RYSKKNREKTLRWVFECIRDGLGYPSIKHDEIGTEQMKEYAKFSLNGNGATDEEAHNWVN  489

Lss     480  VLCMSPGHCGRRKAPKVRTEGGGGSFTSKVFEITLSDGYDWSYADMQMGPHTGDPRDFKT  539
             VLCMSPG  GRRK  K R+EGGG  F +K+ EI+L+DGYDWSYADMQ+GP TGD    K+
5bwe    490  VLCMSPGIHGRRKTQKTRSEGGGSIFPAKLLEISLNDGYDWSYADMQLGPKTGDLSSLKS  549

Lss     540  FDELWNAFRIQNDYVNDMIWRSKDITRKLQMEYLQLPFLSSLDDGCVELGVDGTRLAELP  599
             F+++W AFR Q  Y  ++   +KD++R  +  +LQ+PF+S++DDGC+ELG+D   L+E P
5bwe    550  FEDVWEAFRKQYQYAINLCISTKDVSRYFEQRFLQMPFVSAIDDGCMELGMDACALSEQP  609

Lss     600  NPWLQVHTAIAACNSMIAIKKLVYEDKKYTMAQLIEALHMNWEGYEEMRLDFLNAPKWGN  659
             N W    T I A NS++AIKKLV+E+KKYT+ QL +AL  NWEG+EEMR+DF  APKWGN
5bwe    610  NGWHNPITTIVAANSLVAIKKLVFEEKKYTLEQLSQALKANWEGFEEMRVDFKRAPKWGN  669

Lss     660  DDDYADSLVKAFYEDVLAEKYKRITTYSGAHPLTGSQAVSLYMIIGTFTGPTPDGRFGGE  719
             DDDYAD ++  FYE+++  + ++IT YSG   +   QAV LYM +G+ TGPTPDGRFGGE
5bwe    670  DDDYADGIITRFYEEIIGGEMRKITNYSGGPVMPTGQAVGLYMEVGSRTGPTPDGRFGGE  729

Lss     720  ALDDGGISPMAGTDKNGPTAVLRSMSKLDSSKFKFNLLNQRLSVPLMRSKHGFDIWHAYM  779
             A DDGGISP  GTDK GPTAVLRS+SK+  ++ K NLLNQRLSVP+MRSKHGF+IW++Y+
5bwe    730  AADDGGISPYMGTDKKGPTAVLRSVSKVQKNQ-KGNLLNQRLSVPIMRSKHGFEIWNSYI  788

Lss     780  KTWHDMKIDHVQFNCVTTEEMKAAQVEPEQHEDLIVRVAGYSAKFVDVPTYGQNTIIART  839
             KTWHD+ IDHVQFN V+T+EM+AAQ EPE+H DLIVRV+GYSA+FVD+PTYGQNTIIAR
5bwe    789  KTWHDLNIDHVQFNVVSTDEMRAAQREPEKHHDLIVRVSGYSARFVDIPTYGQNTIIARQ  848

Lss     840  EQKFGPNQFDDLEVEF  855
             EQ F  +  + L VE
5bwe    849  EQDFSASDLEFLNVEI  864
```

2. Alignment of glycine radical enzymes

```
                                10        20        30        40        50        60
Consensus            MEM------- ---Y-GK--- F-PENPAE-D ----IPADE- LHEXLA---- ----NP-STE   30
ABB31773.1           --MS--TVAA SIKYNDKVVD FPLANQEENA ----IADEV- LHENLA---- ----RP-TTE   42
WP_004511527.1       --MS--TVAA SIKYNDKVVD FPLANQEENA ----IADEV- LHENLA---- ----RP-TTE   42
CAO72221.1           --MA--TIAE AVEYRGKIIE FPLEHQEEND ----IPDER- LHEHLA---- ----RP-STQ   42
```

```
CCK78310.1             --MA--TIAE AVEYRGKIIE FPLEHQEEND ----IPDER- LHEHLA---- ----RP-STQ   42
AIS23708.1             ------MALQ SVKYRGKDVN YMPANPAEAD ----IPADE- LHEHLA---- ----NP-STP   40
AAK50372.1             --MNDIASAK VLEYKGKTLN FTPEDPAEAK ----IPSDE- LHEHLQ---- ----KP-STA   44
BAC05501.1             --MNDIVSAK ILEYKGKTLN FTPEDPAEAK ----IPADE- LHEHLQ---- ----KP-STA   44
AAC38454.1             --MNDIVSAK VLEYKGKKLN FTPEDPAEET ----IPADE- LHEHLQ---- ----KP-STA   44
BAD42366.1             ------MTAN VLEYRGKVLN FTPENLAEVN ----IPAEE- LHEHLQ---- ----NP-STE   40
CAI07159.1             --M---TGAQ TMEYKGKVLQ FTPENPAEAD ----IPADE- LHEHLQ---- ----NP-STE   41
WP_011236884.1         --M---TGAQ TMEYKGKVLQ FTPENPAEAD ----IPADE- LHEHLQ---- ----NP-STE   41
CAA05052.1             --M---SDVQ TLEYKGKVVQ FAPENPREAE ----IPADE- LHEHLQ---- ----NP-STE   41
WP_107221981.1         --M---SDVQ TLEYKGKVVQ FAPENPREAE ----IPADE- LHEHLQ---- ----NP-STE   41
LMHFDP_300810          --M---QPVE SVEYKGKVID FPFDNPKEID ----VPPDQ- LAETLV---- ----QP-STE   41
WP_155321809.1         --MT--TVAP SVQYQGQTID FPLENPEEMN ----VSDDR- LTQTLA---- ----SP-STE   42
WP_155306556.1         --MA--RVAE AIEYKGKVIE FPPEYPEELN ----VPTEE- LCETLS---- ----RP-STE   42
WP_014956012.1         --M------- ---------- ---------- ---------- --PTVA---- ----TP-TTE   10
CCK78655.1             --M------- ---------- ---------- ---------- --PTVA---- ----TP-TTE   10
CAO72219.1             --M------- ---------- ---------- ---------- -QENVALKIS EE--TPGSTE   18
CAO72220.1             --M------- ---------- ---------- ---------- -QENVALKIS EE--TPGSTE   18
CAO72222.1             --M------- ---------- ---------- ---------- -QENVALKIA EE--TPGSTE   18
CAO03074.1             --M------- ---------- TATSTLSKTD LKNCETVEE- LRENQQWWW- ----LA-ERE   34
ACL03428.1             --M------- ---------- --VAEPAQ-D ----QSVQE- LEDKQEWWW- ----VA-EKK   27
WP_012610862.1         --M------- ---------- --VAEPAQ-D ----QSVQE- LEDKQEWWW- ----VA-EKK   27
ABH11460.1             MEM------- ---------- --VAEPAQ-D ----QSVQE- LEDKQEWWW- ----VA-EKK   29
ABH11461.1             --M------- ---------- --TAEPAKLD ----VSLQE- HEENQEWWW- ----IA-EKK   28
WP_015946967.1         --M------- ---------- --TAEPAKLD ----VSLQE- HEENQEWWW- ----IA-EKK   28
KFO69021.1             ---------- ---------- --MAEPAK-N ----ISIQE- LEKKQEWWW- ----AA-EKK   26
WP_027352796.1         --M------- ---------- ---ADAKE-- ----AKLQGF LDNNQHWWWA -------EQA   25
AAS96745.1             --M------- --------AA LSSASNTEGQ ----TPANKG YGINWDT--- -------AES   29
sp|P09373.2|PFLB_ECOLI --M------- --SELNEKLA TAWEGFTKGD WQNEVNVRDF IQKNYTPYEG DESFLAGATE   49

                                70         80         90        100        110        120
Consensus              RTKRLK--AR CRWKHAAAGE FVEPGVKAGI ERMRLLTEAH --KASRG--X PEVIRRAKGL   84
ABB31773.1             RTKRLK--AR CRWKHAAAGE FVDAEVRAGI ERMRFITEAH --KASAG--Q PEVIRRALGL   96
WP_004511527.1         RTKRLK--AR CRWKHAAAGE FVDAEVRAGI ERMRFITEAH --KASAG--Q PEVIRRALGL   96
CAO72221.1             RTKRLK--AR CRWKHASAGE FVEKGVTSGI QRMRYITEAH --KKSRG--K PEVIRRALGL   96
CCK78310.1             RTKRLK--AR CRWKHASAGE FVEKGVTSGI QRMRYITEAH --KKSRG--K PEVIRRALGL   96
AIS23708.1             RTARLK--AR CRWKHTSAGE FCEKGVRAGI DRMRLITEAH --KQSEG--K PEVIRRALGL   94
AAK50372.1             RTKRLK--ER CRWKHASAGE FIEKSVTAGI ERMRYLTEAH --KASEG--Q PEVIRRALGL   98
BAC05501.1             RTKRLK--ER CRWKHASAGE FIEKSVTAGI ERMRYLTEAH --KASEG--K PEAIRRALGL   98
AAC38454.1             RTKRLK--ER CRWKHASAGE FIEKSVTAGI ERMRYLTEAH --KASEG--K PEAIRRALGL   98
BAD42366.1             RTKRLK--AR CRWKHASAGE FIEKGVTAGI ERMRHITEAH --KASVG--K PEVIRRALGL   94
CAI07159.1             RTRRLK--AR CRWKHASAGE FCEKGVTAGI ERMRLLTESH --WDTRG--K PEPIRRALGL   95
WP_011236884.1         RTRRLK--AR CRWKHASAGE FCEKGVTAGI ERMRLLTESH --WDTRG--K PEPIRRALGL   95
CAA05052.1             RTRRLK--AR CRWKHAAAGE FCEKGVTAGI ERMRLLTESH --WATRG--E PEPIRRAHGL   95
WP_107221981.1         RTRRLK--AR CRWKHAAAGE FCEKGVTAGI ERMRLLTESH --WATRG--E PEPIRRAHGL   95
LMHFDP_300810          RTRRLK--AR CRYKHTAAGE FVDPNTKGGI ERMRYYTEAH --RASEG--E IQVLRRAKCL   95
WP_155321809.1         RTKRLK--AR CRFKHTAAGE FVNPDLKGGI ERARYFTEGF --KNAGG--K PYILRRAESI   96
WP_155306556.1         RTKRLK--AR CRFKHTAAGE FVDPNLKAGI ERMRYFTESH --KQTVG--M PNAIRRAHAL   96
WP_014956012.1         RTARLK--NR CRFKHVAGGE YVHAGVRAGV ERARLITQSH --KENVG--E PNCIARARGL   64
CCK78655.1             RTARLK--NR CRFKHVAGGE YVHAGVRAGV ERARLITQSH --KENVG--E PNCIARARGL   64
CAO72219.1             RIQFLY--DR CRWKHVAGGM YMRPEVKVGI ARARLLTESY --KETRG--E SEMIRRAKGL   72
CAO72220.1             RIQFLY--DR CRWKHVAGGM YMRPEVKVGI ARARLLTESY --KETRG--E SEMIRRAKGL   72
CAO72222.1             RIQFLY--DR CRWKHVAGGM YMRPEVKVGI ARARLLTESY --KETRG--E SEMIRRAKGL   72
CAO03074.1             RSARLDYLRK ATWKKGALGG NYFDGIRLDL EYPTLFTEAW --KKYPN--D PSMLRRAKAT   90
ACL03428.1             RSKRLDYLRK SIWKKGALGG NYAPGIKLDL ECATLFTDMW --KFWKY--D PIMMRRAKAI   83
WP_012610862.1         RSKRLDYLRK SIWKKGALGG NYAPGIKLDL ECATLFTDMW --KFWKY--D PIMMRRAKAI   83
ABH11460.1             RSKRLDYLRK SIWKKGALGG NYAPGIKLDL ECATLFTDMW --KFWKY--D PIMMRRAKAI   85
ABH11461.1             RSKRLDYLRK AVWKKGALGG NYAPGIKVDL EGPKLFTDMW --NFWKF--D PIIMRRAKAL   84
WP_015946967.1         RSKRLDYLRK AVWKKGALGG NYAPGIKVDL EGPKLFTDMW --NFWKF--D PIIMRRAKAL   84
KFO69021.1             RSKRLDYLRK AMWKKGAIGG LYPAGLQVDL EECQLDTAKA --RELENAPD PYVVKYAKIF   84
WP_027352796.1         RSPRIDYLRK AVWSKGAKAG AYLPGTKVGA DGPILFAEIF DDEDARS--D PYMTTFSRAL   83
AAS96745.1             RVKELK---- --------DF LLAAPQVMDP ERLQCLLDVY --DEFQG--E PVVYIRAKLL   73
sp|P09373.2|PFLB_ECOLI ATTTL----- --WDKVMEGV KLENRTH--- ---------- ---------A PVDFDTAVAS   80

                               130        140        150        160        170        180
Consensus              ANXLDKSTIV LQDDEFI--- ----VGYHAE DPNMFPLYPE LSYMAVQDYL NSPYS---PQ  134
ABB31773.1             ANILNKSTLV LQQDEFI--- ----IGYHAE DPEMFPLYPE LAYMAVQDYL MSDYS---PQ  146
WP_004511527.1         ANILNKSTLV LQQDEFI--- ----IGYHAE DPEMFPLYPE LAYMAVQDYL MSDYS---PQ  146
CAO72221.1             ANILNKYTLV LQEDEFI--- ----IGYHAE DPNMFPLYPE LAYMAVADYL VSDYA---PQ  146
```

```
CCK78310.1              ANILNKYTLV LQEDEFI--- ----IGYHAE DPNMFPLYPE LAYMAVADYL VSDYA---PQ  146
AIS23708.1              KNILDKMTIV VQQDEFI--- ----IGHNAE DPNMFPLYPE LSNLATQDFL MSDYA---PQ  144
AAK50372.1              ANVLNKSTLV LQEDEFI--- ----VGYHAE DPNMFPLYPE LSHMAVQDYL RSDYS---PQ  148
BAC05501.1              ANVLNKSTLV LQEDEFI--- ----VGYHAE DPNMFPLYPE LSHMAVQDYL RSDYS---PQ  148
AAC38454.1              ANVLNKSTLV LQEDEFI--- ----VGYHAE DPNMFPLYPE LSHMAVQDYL RSDYS---PQ  148
BAD42366.1              KNILEKGTLC LQPDEFI--- ----VGYHAE DPNMFPLYPE LSYMAVQDYL LSDYA---PQ  144
CAI07159.1              KNILDKCTLV LQPDEFI--- ----VGYHAE DPNMFPLYPE LSYMAVQDYL KSKYS---PQ  145
WP_011236884.1          KNILDKCTLV LQPDEFI--- ----VGYHAE DPNMFPLYPE LSYMAVQDYL KSKYS---PQ  145
CAA05052.1              KNILDKSTLV LQTDEFI--- ----VGYHAE DPNMFPLYPE LSYMAVQDYL KSKYS---PQ  145
WP_107221981.1          KNILDKSTLV LQTDEFI--- ----VGYHAE DPNMFPLYPE LSYMAVQDYL KSKYS---PQ  145
LMHFDP_300810           ANFLNKMTLV IQEDELV--- ----VGSNAE HPDWIPLHPE TSFVNVIDFV NSPYC---PE  145
WP_155321809.1          ANVLNKTTIV LQEDELI--- ----IGFNAE HPEKFPLFPE TSHLNVIDFI NSPYC---PE  146
WP_155306556.1          ANLLNKCTLV LQEDEFI--- ----VGFNAE HPDKIPLYPE TSYMTVADYI QSRNC---PE  146
WP_014956012.1          EKILKNITIH IQDDELI--- ----VGANTE HPDYFPMYPE LSYFATVDMV ESQYC----D  113
CCK78655.1              EKILKNITIH IQDDELI--- ----VGANTE HPDYFPMYPE LSYFATVDMV ESQYC----D  113
CAO72219.1              DHVLENYPIF INDEEFI--- ----VGDAAE NPDTLAIFPE MGFFPTIDIV EDPELM-DDD  124
CAO72220.1              DHVLENYPIF INDEEFI--- ----VGDAAE NPDTLAIFPE MGFFPTIDIV EDPELM-DDD  124
CAO72222.1              DHILENYPIF INDEEFV--- ----VGDAAE NPDTLAIFPE MGFFPTIDIV EDPELM-DDD  124
CAO03074.1              AYVLDNISIF ITDSAQL--- ----VGYVGS APHTIAWRVD GASTVNSEVY NEPGIH--AE  141
ACL03428.1              AHVLDKKTIF ITDHAQL--- ----VGYFGS LPNTIMWRVD GASMVNEEAY NEPGIM--PE  134
WP_012610862.1          AHVLDKKTIF ITDHAQL--- ----VGYFGS LPNTIMWRVD GASMVNEEAY NEPGIM--PE  134
ABH11460.1              AHVLDKKTIF ITDHAQL--- ----VGYFGS LPNTIMWRVD GASMVNEEAY NEPGIM--PE  136
ABH11461.1              AHVFDNISIF ITDHSQI--- ----VGYWGS APHTISWRVD GASIVNEELY NEPGIM--PE  135
WP_015946967.1          AHVFDNISIF ITDHSQI--- ----VGYWGS APHTISWRVD GASIVNEELY NEPGIM--PE  135
KFO69021.1              AHYLDNKTIF ITDKAQL--- ----VSYVGS LPNTIGWNPT TASMVNLEVL NDSSAL--PE  135
WP_027352796.1          ENTNKKLPVY IVDNSRI--- ----VGCCAA APHEVFWVPN LSFGLNEDIF NDRDELVDMD  136
AAS96745.1              ERVLLRKKIF LDGNPIVGTL TGVRAGVYAY PEWNVSWIKE EMQMAKMASL GEMKI---PQ  130
sp|P09373.2|PFLB_ECOLI  TITSHDAGYI NKQLEKI--- ----VGLQTE APLKRALIPF GGIKMIEGSC KAYNR-----  128

                                  190        200        210        220        230        240
Consensus               PADEA----A EINDYWKPYS LQDKCEPYFD PEDLXR---M YQVST-MEAP S-FAXGYNSI  185
ABB31773.1              PKEEA----A EINEYWKKYS MQAKGERYFT QEELLQ---M YQVST-MEAP G-FATGYNSI  197
WP_004511527.1          PKEEA----A EINEYWKKYS MQAKGERYFT QEELLQ---M YQVST-MEAP G-FATGYNSI  197
CAO72221.1              PAEEA----K EIMEYWKPYS MQGKCEGYFD PEDLMR---M YQVST-MEAP G-FATGYNSI  197
CCK78310.1              PAEEA----K EIMEYWKPYS MQGKCEGYFD PEDLMR---M YQVST-MEAP G-FATGYNSI  197
AIS23708.1              PVDEG----T EINDYWRQHS LQAKCMSYFD PVDLAR---M FKFSS-MESP P-FVSAYNSI  195
AAK50372.1              PADEA----A AINDYWKPHS LQSKCQPYFD PADLGR---M YQVSS-MEAP P-FASGYNSI  199
BAC05501.1              PADEA----A AINEYWKPHS LQSKCQPYFD PADLGR---M YQVSS-MEAP S-FASGYNSI  199
AAC38454.1              PADEA----A AINEYWKPHS LQSKCQPYFD PADLGR---M YQVSS-MEAP S-FASGYNSI  199
BAD42366.1              PTSEA----A EINDYWKPYS LQAKCSPYFD PTDLGR---M FQVSS-MEAP S-FASGYNSI  195
CAI07159.1              PAKEA----Q EIVDYWKPFS LQARCEPYFD PVDLRR---G YQVST-IEGP V-FASGYNSV  196
WP_011236884.1          PAKEA----Q EIVDYWKPFS LQARCEPYFD PVDLRR---G YQVST-IEGP V-FASGYNSV  196
CAA05052.1              PAKEA----Q EIVDYWKPFS LQARCEPYFD PVDLHR---G YQVST-IEGP V-FATGYNSV  196
WP_107221981.1          PAKEA----Q EIVDYWKPFS LQARCEPYFD PVDLHR---G YQVST-IEGP V-FATGYNSV  196
LMHFDP_300810           EKEEA----I EYAQYWARHS IMYKGERYFT REELDQ---Q YQFST-MEAP T-FANAYNST  196
WP_155321809.1          EKEEA----V ELVEYWEKYG LVTKGARYFS ELELQQ---M YQFST-MEAP A-FANAYNST  197
WP_155306556.1          EKEEA----I EYNEYWKDHS IQALGQRYFT QEELLQ---M YQFTT-MEPP A-FANAYNSV  197
WP_014956012.1          HKDEM----R EIAEYWRPYT IQTKGEKYFT PEEIGV---M YSATT-VQPP M-FVTAFSSI  164
CCK78655.1              HKDEM----R EIAEYWRPYT IQTKGEKYFT PEEIGV---M YSATT-VQPP M-FVTAFSSI  164
CAO72219.1              IRDEA----R EIAMFWKPLG LQDKCMPYYD QHEIDI---A TPWTI-VDVP P-FIANYMSV  175
CAO72220.1              IRDEA----R EIAMFWKPLG LQDKCMPYYD QHEIDI---A TPWTI-VDVP P-FIANYMSV  175
CAO72222.1              IRDEA----R EIAMFWKPLG LQDKCMPYYD QHEIDI---A TPWTI-VDVP P-FIANYMSV  175
CAO03074.1              PEAESLKKVA EINSYWNGQT AVDKVGRLID PEDAVK---F FSGAIGWGTP S-SAFGYSGK  197
ACL03428.1              PENESLQKVA ELNDYWAGQT AVDKVARILD PEDAVK---F LSGAIGWGAP S-SAYGYSGK  190
WP_012610862.1          PENESLQKVA ELNDYWAGQT AVDKVARILD PEDAVK---F LSGAIGWGAP S-SAYGYSGK  190
ABH11460.1              PENESLQKVA ELNDYWAGQT AVDKVARILD PEDAVK---F LSGAIGWGAP S-SAYGYSGK  192
ABH11461.1              PEEESLRKVA EINDYWAGQT AVDKVARILD PEDAVK---F LSGAIGWGAP T-SAYGYSGK  191
WP_015946967.1          PEEESLRKVA EINDYWAGQT AVDKVARILD PEDAVK---F LSGAIGWGAP T-SAYGYSGK  191
KFO69021.1              PLDASLKVIN EVAAYWAGKA DADRMMPRVD MTDVMK---V LSGTIGWGSA V-ARLGYSGK  191
WP_027352796.1          KRDEV----E KCLKIMKPYT QQFMAEKVMS KRHKIM---C RTSQTYTGGP HLEGLFYCTS  189
AAS96745.1              ETQELL---E KTYKLWKGRT CIDLNNKMFK EKYGINPAPF AKAGMYYENV S-VASG--SG  184
sp|P09373.2|PFLB_ECOLI  ELDPMI---K KIFTEYRKTH NQGVFDVY-- TPDILR---C RKSGVLTGLP --DAYGRGRI  178

                                  250        260        270        280        290        300
Consensus               VPPYETVLED GLLARIELAE ENIEKAKA-- -EM-RKFPWN GPEGLPWIDK IDNWEAMIIA  241
ABB31773.1              SPPYETVLQD GLLKRIEMAH EKIEHAKR-- -EM-QKIPWD ATTGLDWIAK IDVWKAMIIA  253
WP_004511527.1          SPPYETVLQD GLLKRIEMAH EKIEHAKR-- -EM-QKIPWD ATTGLDWIAK IDVWKAMIIA  253
CAO72221.1              VPPYETILED GLLKRIEMAE ANIKAAKE-- -DL-KKTPWD ATKGLKWIPM IDNWEAMIIA  253
```

```
CCK78310.1              VPPYETILED GLLKRIEMAE ANIKAAKE-- -DL-KKTPWD ATKGLKWIPM IDNWEAMIIA  253
AIS23708.1              VPPYETVLED GLLARIKLAE GNIAHAKE-- -EM-GKFPWD GTKGLVWLDK IDNWEAMIIA  251
AAK50372.1              VPPYETVLED GLLARIKLAE KHIAEAQA-- -DM-STFPWN GTKGLDNIAK IDHWKAMVIA  255
BAC05501.1              VPPYETVLED GLLARIKLAE KHIAEAQG-- -DM-STFPWN GTKGLDNIAK IDNWKAMVIA  255
AAC38454.1              VPPYETVLED GLLARIKLAE KHIAEAQA-- -DM-STFPWN GTKGLDNIAK IDNWKAMVIA  255
BAD42366.1              VPPYETVLED GLLARVKLAK SHIEQAQA-- -EM-GAFPWN GSKGLEWIEK IDNWQAMIIA  251
CAI07159.1              IPPYETILED GLLARIALAE KNIEHARA-- -EM-EKFPWN APTGLEWIDK IDNWEAMVIA  252
WP_011236884.1          IPPYETILED GLLARIALAE KNIEHARA-- -EM-EKFPWN APTGLEWIDK IDNWEAMVIA  252
CAA05052.1              IPPYETVLED GLQARIALAE EKIEHARA-- -EM-EKFPWH APSGLEWIDK IDNWKAMVIA  252
WP_107221981.1          IPPYETVLED GLQARIALAE EKIEHARA-- -EM-EKFPWH APSGLEWIDK IDNWKAMVIA  252
LMHFDP_300810           VPPYETIMED GLLKRIAMCK ENIAKAKA-- -EL-AAPDWN GPERLPLCDK IDQWDAMIMA  252
WP_155321809.1          VPPYETIMED GLNKRIAMAE ENIARAKA-- -EL-AAADWN GPERLPLLDK IDTWEAMIIV  253
WP_155306556.1          CPPYETVLED GLLKRIEIAE QNKQNAMN-- -EM-QSPAWN APQRLPLMEK IDNWDAMIIA  253
WP_014956012.1          VPTYEAVLED GLIKRIEEVE KKIADANA-- -EM-RKSPWN GQENLHYLDK IDQWNAMLIA  220
CCK78655.1              VPTYEAVLED GLIKRIEEVE KKIADANA-- -EM-RKSPWN GQENLHYLDK IDQWNAMLIA  220
CAO72219.1              CPAYMSVLED GLLGRIKSAE ENIEKAFV-- -KL-RAYPWN GPENMPLMDQ IDVWRAMIIA  231
CAO72220.1              CPAYMSVLED GLLGRIKSAE ENIEKAFV-- -KL-RAYPWN GPENMPLMDQ IDVWRAMIIA  231
CAO72222.1              CPAYMSVLED GLLGRIKSSE ENIAKAFV-- -KL-RAYPWN GPENMPLMDQ IDVWRSMIIA  231
CAO03074.1              NFEYFMKGDR AFSQIIAEID EKIDEAEE-- -AT-IGTP-- SPHILPLYDK LNNWHAMKLV  251
ACL03428.1              DYEYLFAGRR GFEDIIEEIN AAIEKAED-- -KT-VGVP-- GPEILDIYDR LQNWDAMILV  244
WP_012610862.1          DYEYLFAGRR GFEDIIEEIN AAIEKAED-- -KT-VGVP-- GPEILDIYDR LQNWDAMILV  244
ABH11460.1              DYEYLFAGRR GFEDIIEEIN AAIEKAED-- -KT-VGVP-- GPEILDIYDR LQNWDAMILV  246
ABH11461.1              NYEYYLKGER GFEDIIADIE DHIAEAEE-- -KT-IGTP-- GPDILPIYDR IQNWEAMITV  245
WP_015946967.1          NYEYYLKGER GFEDIIADIE DHIAEAEE-- -KT-IGTP-- GPDILPIYDR IQNWEAMITV  245
KFO69021.1              DYEYIMTGKR GFEDVIAEID ENMDKADA-- -QAHMPAA-- NKEIGALYDK MNTWEAMKIT  246
WP_027352796.1          QFTYYS---K GFNAIIEEID AGLKEASNVL YKM-GAVP-N FPDEEHYLQR VPVWEAMKRT  244
AAS96745.1              IADYPLVLNK GLRWLADDVR ARFEAC---- ---------- -PTTLANKEK HDLYRAMLVT  229
sp|P09373.2|PFLB_ECOLI  IGDYRRVALY GIDYLMK--- ---------- ---------- --------DK LAQFTSLQAD  207

                                  310        320        330        340        350        360
Consensus               DKAVIAWARR YARLAKIVAE NFETD----- -PKRKEELLE IADICQRVPA EPARGLKDAM  295
ABB31773.1              DEAVINWARR HARLAKIVAE NFETN----- -PARKEELLE IAEISHRVPA EPCKGLKDAF  307
WP_004511527.1          DEAVINWARR HARLAKIVAE NFETN----- -PARKEELLE IAEISHRVPA EPCKGLKDAF  307
CAO72221.1              DKAVIAWARR HARLAKFVAE NIETD----- -PKRKEELLE IADINQRIPA EPCKGLKDAF  307
CCK78310.1              DKAVIAWARR HARLAKFVAE NIETD----- -PKRKEELLE IADINQRIPA EPCKGLKDAF  307
AIS23708.1              CKAVIAWARR HARMCRIVAE NFETD----- -PKRQTELLE IAEICQRIPA EPCKGLKDAF  305
AAK50372.1              CKAVISWARR QGRLCRIVAE NFETD----- -PKRQAELLE VADICHRVPA EPCKGLKDAM  309
BAC05501.1              CKAVISWARR QARLCRIVAE NFETD----- -PKRQAELLE IADICHRIPA EPCKGLKDAM  309
AAC38454.1              CKAVISWARR QGRLCKIVAE NFETD----- -PKRQAELLE IADICQRIPA EPCKGLKDAM  309
BAD42366.1              CEAVISWARR HARMCKIVAE KFEAD----- -PKRRAELLE IADICQRVPA EPCKGLKDAF  305
CAI07159.1              CKAVIAWARR HARLCKIVAE RFETD----- -PKRKAELLE IADICQRVPA EPARGLKDAM  306
WP_011236884.1          CKAVIAWARR HARLCKIVAE RFETD----- -PKRKAELLE IADICQRVPA EPARGLKDAM  306
CAA05052.1              CKAVIAWARR HARLCKIVAE HFETD----- -PKRKAELLE IADICQRMPA EPARGLKDAM  306
WP_107221981.1          CKAVIAWARR HARLCKIVAE HFETD----- -PKRKAELLE IADICQRMPA EPARGLKDAM  306
LMHFDP_300810           DEAVIAWAQR YGRLAKIIAE NFESD----- -PKRKTELLE IADICHRTPA EPCKGLRDAM  306
WP_155321809.1          DQAVINWAQR HGRMCRIVAE KFESD----- -AKRQAELME IADICQRVPA EPCRGLRDAM  307
WP_155306556.1          DKAVISWARR HGRLCKIVAE NFETD----- -PKRQEELIE IAGICHRMPA EPAKGLRDAM  307
WP_014956012.1          MKAVVAWAQR YARLAKIMAE NFLTD----- -PKRKEELLE IADICRHVPA YPARGLKDAM  274
CCK78655.1              MKAVVAWAQR YARLAKIMAE NFLTD----- -PKRKEELLE IADICRHVPA YPARGLKDAM  274
CAO72219.1              DKAVIKWARR YSRLAKIVAE NFDLSDSVQG AEERKNELLE ISDICYRMPA EPAKGFKDAM  291
CAO72220.1              DKAVIKWARR YSRLAKIVAE NFDLSDSVQG AEERKNELLE ISDICYRMPA EPAKGFKDAM  291
CAO72222.1              DKAVIKWARR YGRLAKIIAE NFDLSDSVLG AEGRKNELLE ISDICYRMPA EPAKGFKDAM  291
CAO03074.1              LEAAIRFAGR YARLARVMAA K-ETD----- -EQRKKELLR VAETCERVPA NPPRNLQESL  304
ACL03428.1              LEAGIRHAKR YARLARTMAE NMETD----- -EKRREELLK IAETCERVPA RAPRNLQESL  298
WP_012610862.1          LEAGIRHAKR YARLARTMAE NMETD----- -EKRREELLK IAETCERVPA RAPRNLQESL  298
ABH11460.1              LEAGIRHAKR YARLARTMAE NMETD----- -EKRREELLK IAETCERVPA RAPRNLQESL  300
ABH11461.1              LEAAIRFAKR YARLARTMAE HLETD----- -EKRKEELLR IAETCERVPA KAPRNLQESF  299
WP_015946967.1          LEAAIRFAKR YARLARTMAE HLETD----- -EKRKEELLR IAETCERVPA KAPRNLQESF  299
KFO69021.1              LEAGIRHAQR YARLARIIAE NFETD----- -NKRKEELLQ IANCCERVPA KPPRTLQESL  300
WP_027352796.1          LQSEILYAKR LARLARIISE NFETD----- -SERKQELLD ISERCEWVPA NRPRNFPEAL  298
AAS96745.1              FEAVIAHSHR YAELAEKTAA E-ESD----- -PKAKAELLE IAEICRRVPE YPARNFREAI  282
sp|P09373.2|PFLB_ECOLI  LENGVNLEQT -IRLREEIAE Q--------- -HRALGQMKE MAAKYGYDIS GPATNAQEAI  256

                                  370        380        390        400        410        420
Consensus               QAKWFTYLIC HAIERYASGY AQKEDXLLWP YYKASVIDKT FQPMTHEDAV ELVEMERLKV  355
ABB31773.1              QAKWYTYLIC HAIDRYASGY AQKEDEMLEP YYNISVKEKS FQPMTHTDVV EMVEMERLKI  367
WP_004511527.1          QAKWYTYLIC HAIDRYASGY AQKEDEMLEP YYNISVKEKS FQPMTHTDVV EMVEMERLKI  367
CAO72221.1              QAKWYTFLIC HAIDRYASGF AQTEDTMLYP YYKASVIDKT FQPMTHSEAV EMVEMERLKI  367
```

```
CCK78310.1             QAKWYTFLIC HAIDRYASGF AQTEDTMLYP YYKASVIDKT FQPMTHSEAV EMVEMERLKI  367
AIS23708.1             QAKWFTFLIC HSLERYASGF AHQEDKLLWP YYKASVIDRT AQPMTRKEAV EWVEMERLKV  365
AAK50372.1             QAKFFTFLIC HAIERYASGY AQKEDTLLWP YYKASVIDKK FQPMDHMGAV ELVEMERLKI  369
BAC05501.1             QAKFFTFLIC HAIERYASGY AQKEDTLLWP YYKASVIDKK FQPMDHMGAV ELVEMERLKI  369
AAC38454.1             QAKFFTFLIC HAIERYASGY AQKEDTLLWP YYKASVVDKK FQPMSHMDAV ELVEMERLKI  369
BAD42366.1             QAKWFTYLIC HAIDRYASAT AHKEDTLLWP YYKASVVDKS FQPMTYENAV EWVEMERLKI  365
CAI07159.1             QAKWFTFLIC HAIERYASGY AQKEDSLLWP YYKASVIDKT FQPMEHKDAV ELIEMERLKV  366
WP_011236884.1         QAKWFTFLIC HAIERYASGY AQKEDSLLWP YYKASVIDKT FQPMEHKDAV ELIEMERLKV  366
CAA05052.1             QSKWFTFLIC HAIERYASGF AQKEDSLLWP YYKASVIDKT FQPMEHKDAV ELIEMERLKV  366
WP_107221981.1         QSKWFTFLIC HAIERYASGF AQKEDSLLWP YYKASVIDKT FQPMEHKDAV ELIEMERLKV  366
LMHFDP_300810          QVKWFTYLVC HSLEAYASGY AHKEDKVLWP YYKMSVIDKS FQPMTHADAV ELVECERLKV  366
WP_155321809.1         QVKWFTYLVC HAIETYASGY AHKEDKVLWP YYQVSVIDQS FQPMTHADVV ELVECERLKV  367
WP_155306556.1         QAKWFTYLIS HSLENYASGY AHKEDKLLYP YYKTSVIDKS FQPMTYEDAV ELVECERLKV  367
WP_014956012.1         QSKWFTYLLC HSIERYSSGY GQKEDKMLWP YFQKSVIEKT EQPMTREEAV ELFECERLKV  334
CCK78655.1             QSKWFTYLLC HSIERYSSGY GQKEDKMLWP YFQKSVIEKT EQPMTREEAV ELFECERLKV  334
CAO72219.1             QSKWFVYLVC HSLERYSSGY AHLEDRLMWP YYKASVIDKT AQPMTRDEAI QLVELERLKV  351
CAO72220.1             QSKWFVYLVC HSLERYSSGY AHLEDRLMWP YYKASVIDKT AQPMTRDEAI QLVELERLKV  351
CAO72222.1             QSKWFVYLVC HSLERYSSGY AHLEDRLMWP YYKASVIDKT AQPMTRDEAI ELIECERLKV  351
CAO03074.1             QYEHFVQVLA RY-EAHEGAW PSRPDYYHGP LYAKDV--EV EKNITESEAI DLVGEYMIRC  361
ACL03428.1             QYDHFIQIFA RT-EAHEGAW PARPDYYHGP YYDKDV--NV DKTLTKEDAL DLVGEFMIRA  355
WP_012610862.1         QYDHFIQIFA RT-EAHEGAW PARPDYYHGP YYDKDV--NV DKTLTKEDAL DLVGEFMIRA  355
ABH11460.1             QYDHFIQIFA RT-EAHEGAW PARPDYYHGP YYDKDV--NV DKTLTKEDAL DLVGEFMIRA  357
ABH11461.1             QMDMLIQTMC RF-EASEGAW PARPDYYHGP FYEKDV--LQ DKTLTEEEAT DLIGEFMIRA  356
WP_015946967.1         QMDMLIQTMC RF-EASEGAW PARPDYYHGP FYEKDV--LQ DKTLTEEEAT DLIGEFMIRA  356
KFO69021.1             QYDLFIQNFS RT-EAVEGSW PARPDYYHGQ YYDKDV--NI DKRITKEEAM DLVGEFLIRA  357
WP_027352796.1         QFEHFQTMAR KR-EKPDGAW PAHPDWWFGD WFDADM---K SGYITREDAV DYVGEYLIRS  354
AAS96745.1             QSFWFIHLAI ETEQMACATS PGRYGGQYMYP FYKKDI---E EGNLTREQVL TLLKFQWIKH  339
sp|P09373.2|PFLB_ECOLI QWTYFGYLAA VKSQNGAAMS FGRTSTFLDV YIERDL---K AGKITEQEAQ EMVDHLVMKL  313

                                430       440       450       460       470       480
Consensus              SEHGAGKSRA YREIFPGSND LFILTLGGTN PDGSDACNDM TDAILEAARX I-RTTEPSIV  414
ABB31773.1             SEHGAGKSRA YREIFPGSND LFILTIGGTK PGYVDACSDM TDAILEGARN I-RTTEPSIV  426
WP_004511527.1         SEHGAGKSRA YREIFPGSND LFILTIGGTK PGYVDACSDM TDAILEGARN I-RTTEPSIV  426
CAO72221.1             SEHGAGKSRA YREIFPGSND LFILSIGGTK PDGSDASNEM TNAILEATRN I-RTTEPSIV  426
CCK78310.1             SEHGAGKSRA YREIFPGSND LFILSIGGTK PDGSDASNEM TNAILEATRN I-RTTEPSIV  426
AIS23708.1             SEHGSGKSRA YREAFPGAGD LYIVTIGGTK GDGSDACNDM TDAILEAAKR I-RTTEPSIV  424
AAK50372.1             SEHGAGKSRA YREIFPGSND LFILTVGGTN AKGEDACNDM TDAILEATKR I-RTAEPSIV  428
BAC05501.1             SEHGAGKSRA YREIFPGSND LFILTVGGTN ARGEDACNDM TDAILEAAKR I-RTAEPSIV  428
AAC38454.1             SEHGAGKSRA YREIFPGSND LFILTVGGTN AKGEDACNDM TDAILEAAKR I-RTAEPSIV  428
BAD42366.1             SEHGAGKSRA YREIFPGSND LFILTVGGTL GDGSDACTDM TDAILEGAKR I-RTTEPSIV  424
CAI07159.1             SEHGAGKSRA YREIFPGSND LFILTLGGTN GDGSDACNDM TDAILEAAKR I-RTTEPSIV  425
WP_011236884.1         SEHGAGKSRA YREIFPGSND LFILTLGGTN GDGSDACNDM TDAILEAAKR I-RTTEPSIV  425
CAA05052.1             SEHGAGKSRA YREIFPGSND LFILTLGGTN GDGSDACNDM TDAILEATKR I-RTTEPSIV  425
WP_107221981.1         SEHGAGKSRA YREIFPGSND LFILTLGGTN GDGSDACNDM TDAILEATKR I-RTTEPSIV  425
LMHFDP_300810          SEHGCAKNRM YRITFAGSND LFILSVGGLN SDGTDGCNDM TDAILEAARN I-RTTEPSIG  425
WP_155321809.1         SEHGCGKNRA YRSAFAGSND LFILTLGGTN KDGSDGCNDM TDAILQAAKN I-RTTEPSIG  426
WP_155306556.1         SEHGCGKNRA YREAFPGSND LFILTLGGVN VDGTDGCNDM TDAILDAAKN I-RTTEPSIG  426
WP_014956012.1         SEHGSTKGRQ LREFFAGSND LFILTLGGIN PDGSDASNDC TNCILEAASS I-VTTEPSIS  393
CCK78655.1             SEHGSTKGRQ LREFFAGSND LFILTLGGIN PDGSDASNDC TNCILEAASS I-VTTEPSIS  393
CAO72219.1             CERGVAKGRA HREGQPGAND LHIITIGGLD ENGNDATNDL TNVILEASLN I-RTPEPSLG  410
CAO72220.1             CERGVAKGRA HREGQPGAND LHIITIGGLD ENGNDATNDL TNVILEASLN I-RTPEPSLG  410
CAO72222.1             CERGVAKGRA HREGQPGAND LHIITIGGLD ENGNDATNDL TNAILEASLS V-RTPEPSLG  410
CAO03074.1             SEYGSFSPRY MREGLQGVTG TFVWTLGGVN QDGTDACNGM TIALLKAARL V-RVANPTFG  420
ACL03428.1             YEVGGFAPRW AREGLQGITG TWVWTLGGVN KDGSDACNDL TVAFLQAARL V-RVSNPTFG  414
WP_012610862.1         YEVGGFAPRW AREGLQGITG TWVWTLGGVN KDGSDACNDL TVAFLQAARL V-RVSNPTFG  414
ABH11460.1             YEVGGFAPRW AREGLQGITG TWVWTLGGVN KDGSDACNDL TVAFLQAARL V-RVSNPTFG  416
ABH11461.1             YEVGGFGPRW AREGMQGITG TWVWTIGGVK PDGSDACNAL TTAFLRTARL I-RVSNPTFA  415
WP_015946967.1         YEVGGFGPRW AREGMQGIEG TWVWTIGGVK PDGSDACNAL TTAFLRTARL I-RVSNPTFA  415
KFO69021.1             AEVSQYKPKW AREGLQGIEG TWVWTLGGVK QDGSDACNDM TIALLQAARL V-RVANPTFS  416
WP_027352796.1         YEYGSCRNRQ WRELMTGDPG PYVWTFGGMR PDGTMDYRDL INVFMEAARY V-RCVSPTFA  413
AAS96745.1             LELGEYQGAS YAMTLSGHTG QSI-TIGGVD ANGDDASTEL EEVLLDTQIQ M-KNIQPTLT  397
sp|P09373.2|PFLB_ECOLI RMVRFLRTPE YDELFSGDPI WATESIGGMG LDGRTLVTKN SFRFLNTLYT MGPSPEPNMT  373

                                490       500       510       520       530       540
Consensus              FRYSPKXREK TLRLVFECIR DGLGYPSIKN DELGTQQLLE Y-------NG ATPEEARTWV  467
ABB31773.1             FRWHPVGREK TKRLVFECIR DGLGYPSIKH DVIGTEQLKY YSQFSKNNNG ATDDEAHYWG  486
WP_004511527.1         FRWHPVGREK TKRLVFECIR DGLGYPSIKH DVIGTEQLKY YSQFSKNNNG ATDDEAHYWG  486
CAO72221.1             FKYSDKSNDK TKRLVFECIR DGLGYPSIKH NEIAVEQLKY YSQFSKEGNG ATDDEAHNWA  486
```

```
CCK78310.1             FKYSDKSNDK TKRLVFECIR DGLGYPSIKH NEIAVEQLKY YSQFSKEGNG ATDDEAHNWA  486
AIS23708.1             FRYSKKNRPE TLRLVFECIR DGLGYPSIKN DDIGTAQMLE YSKYSLNGNG ATPEEAHDWA  484
AAK50372.1             FRYSKKSREK TLRWVFECIR DGLGYPSIKH DEIGTAQMKE YAKFSLNGNG ATDEEAHNWV  488
BAC05501.1             FRYSKKNREK TLRWVFECIR DGLGYPSIKH DEIGTEQMKE YAKFSLNGNG ATDEEAHNWV  488
AAC38454.1             FRYSKKNREK TLRWVFECIR DGLGYPSIKH DEIGTEQMKE YAKFSLNGNG ATDEEAHNWV  488
BAD42366.1             FRYSKKSRAK TLRWVFECVR DGLGYPSIKH DEIGTAQMLE YGAYSLTGNG ATPEEAHNWV  484
CAI07159.1             FRYSKKNRAK TLRWVFECIR DGLGYPSIKN DDLGIQQLLE MAKYSRNGNG VTPEEAHYWV  485
WP_011236884.1         FRYSKKNRAK TLRWVFECIR DGLGYPSIKN DDLGIQQLLE MAKYSRNGNG VTPEEAHYWV  485
CAA05052.1             FRYSKKNRAK TLRWVFECIR DGLGYPSIKH NELGVQQMLE MAKYSRNGNG ATPEEAHYWV  485
WP_107221981.1         FRYSKKNRAK TLRWVFECIR DGLGYPSIKH NELGVQQMLE MAKYSRNGNG ATPEEAHYWV  485
LMHFDP_300810          FRWNKIGREK TKRLVFECIR DGLGYPSIKN DELNTDQLIN A-------FG ATPEEARDWA  478
WP_155321809.1         FRWSPKGRKK TKRLVFECIR DGLGFPSIKN DELATEQLMA Q-------FG ASEEEARDWA  479
WP_155306556.1         FRWNKIGREK TKRKVFECVR DGLGYPSIKN DEIAIEQLQK N-------FG ATLEEARDWA  479
WP_014956012.1         FRWNEIGNIE TKKRVFDCVK KGFGFPSIKN DELNTQQLVK Y-------FN VPEEVARDWA  446
CCK78655.1             FRWNEIGNIE TKKRVFDCVK KGFGFPSIKN DELNTQQLVK Y-------FN VPEEVARDWA  446
CAO72219.1             FRYSPKINAK TRRLVFENIA AGFGFPSIKH EEKNTRQMLE H-------YK VPPDEAAHWA  463
CAO72220.1             FRYSPKINAK TRRLVFENIA AGFGFPSIKH EEKNTRQMLE H-------YK VPPDEAAHWA  463
CAO72222.1             FRYSPKINAK TRRLVFENIA AGFGFPSIKH EEKNTRQMLE H-------YK VPPDEAAHWA  463
CAO03074.1             FRWHPKVSNE VLRECFECIR QGLGYPTLRN DPVLIQNTMH W-------YG HPLEEARTWV  473
ACL03428.1             FRWHPKVKDE VLRECFECIR HGLGYPSMRN DPLLIQNAMH W-------HG HPLEEARTWV  467
WP_012610862.1         FRWHPKVKDE VLRECFECIR HGLGYPSMRN DPLLIQNAMH W-------HG HPLEEARTWV  467
ABH11460.1             FRWHPKVKDE VLRECFECIR HGLGYPSMRN DPLLIQNAMH W-------HG HPLEEARTWV  469
ABH11461.1             FRWHPKVSDE VMRECFECIR HGLGYPSFRH DPILVANCMN W-------HG HPVEEARTWV  468
WP_015946967.1         FRWHPKVSDE VMRECFECIR HGLGYPSFRH DPILVANCMN W-------HG HPVEEARTWV  468
KFO69021.1             FRWHPKVKEE VLRECFECIR QGLGYPSMRN DPLLIANSMH W-------HG HPIEEARTWV  469
WP_027352796.1         LRYNKEMPED ILKNCFDCIR HGLGYPNIRN DQVLIKANMF W-------SN TPEEEARTWV  466
AAS96745.1             LLYHPKLKDS YMKKVVECIR GGSGQPQILN NNVVIQRNLA R--FAQYEGG ITLEDARNCG  455
sp|P09373.2|PFLB_ECOLI ILWSEKLPLN FKKFAAKVSI DTSSL-QYEN DDLM------ ---------- RPDFNNDDYA  416

                                 550        560        570        580        590        600
Consensus              NVLCMSPGPT GRRKTQKTRS EGGGSIFPAK MLEIXLNNGY DWSYADMQMG PXTGDAT-DF  526
ABB31773.1             LVLCMSPGVC GRRKTHKTRS EGGGSIFPAK MMEIVLADGF DWSYSGMQLG PHTGDPT-TF  545
WP_004511527.1         LVLCMSPGVC GRRKTHKTRS EGGGSIFPAK MMEIVLADGF DWSYSGMQLG PHTGDPT-TF  545
CAO72221.1             NVLCMSPGLC GRRKTQKTRS EGGGSIFPAK ILEVTLNDGY DWSYADMQLG PQTGKAE-DF  545
CCK78310.1             NVLCMSPGLC GRRKTQKTRS EGGGSIFPAK ILEVTLNDGY DWSYADMQLG PQTGKAE-DF  545
AIS23708.1             NVLCMSPGLV GRRKTQKTRS EGGGGIFPAK IFEISLTNGY DWSYSDFQMG PETGDAR-NF  543
AAK50372.1             NVLCMSPGLH GRRKTQKTRS EGGSSVFPAK VLEITLNDGY DWSYADMQLG PKTGELS-SL  547
BAC05501.1             NVLCMSPGIH GRRKTQKTRS EGGGSIFPAK LLEITLNDGY DWSYADMQLG PKTGDLT-SL  547
AAC38454.1             NVLCMSPGIH GRRKTQKTRS EGGGSIFPAK LLEISLNDGY DWSYADMQLG PKTGDLS-SL  547
BAD42366.1             NVLCMSPGLA GRRKTQKTRS EGGGSIFPAK LLEITLNDGY DWSYADMQLG PKTGQAT-QF  543
CAI07159.1             NVLCMAPGVA GRRKAQKTRS EGGSAIFPAK LLEITLSNGY DWSYADMQMG PETGHAK-DF  544
WP_011236884.1         NVLCMAPGVA GRRKAQKTRS EGGSAIFPAK LLEITLSNGY DWSYADMQMG PETGHAK-DF  544
CAA05052.1             NVLCMAPGLA GRRKAQKTRS EGGSAIFPAK LLEITLNNGY DWSYADMQMG PETGYAK-DF  544
WP_107221981.1         NVLCMAPGLA GRRKAQKTRS EGGSAIFPAK LLEITLNNGY DWSYADMQMG PETGYAK-DF  544
LMHFDP_300810          LVLCMSPGHC GRRKAPKVRT EGGGGSFTSK VFEITLSDGY DWSYADMQMG PHTGDPR-DF  537
WP_155321809.1         LVLCMSPGHC GRRKAQKVRT EGGGGSFTAK VFEITLSDGF DWSFSGFQMG PKSGDPR-DF  538
WP_155306556.1         LQLCMSPGHV GRRKTQKART EGGGSLLPAK MFEITLSDGY DWSHSDMQLG PKTGDPR-EF  538
WP_014956012.1         LVLCMSPGIT GRRGTQKTRS EGGSDVYPAK VMEIALTNGF DEFFTNIQLG PETGNGE-DF  505
CCK78655.1             LVLCMSPGIT GRRGTQKTRS EGGSDVYPAK VMEIALTNGF DEFFTNIQLG PETGNGE-DF  505
CAO72219.1             LVLCMAPGVS KRRGLQKTRT EGGGLIWIDK CCEIAFYDGF DHSFANIQTG PKTGDAT-KF  522
CAO72220.1             LVLCMAPGVS KRRGLQKTRT EGGGLIWIDK CCEIAFYDGF DHSFANIQTG PKTGDAT-KF  522
CAO72222.1             LVLCMAPGVG KRRGLQKTRT EGGGLIWIDK CCEIAFYDGF DHSFANIQTG PKTGDAT-KF  522
CAO03074.1             HMACMSPNPT TKHGTSPFRM ASA-TMNSAK TIEYVLHNGY D-RVVNMQMG PKTGDAR-EI  530
ACL03428.1             HQACMSPCPT TKHGVQPMRM ASA-TANCAK MVEYALHNGY D-HVVGMQMG PETGDAA-KF  524
WP_012610862.1         HQACMSPCPT TKHGVQPMRM ASA-TANCAK MVEYALHNGY D-HVVGMQMG PETGDAA-KF  524
ABH11460.1             HQACMSPCPT TKHGVQPMRM ASA-TANCAK MVEYALHNGY D-HVVGMQMG PETGDAA-KF  526
ABH11461.1             HQACMSPCPT TKNGVQPFRM ASA-TANCAK MVEYALHNGY D-HVVGMQMG PQTGDAR-TF  525
WP_015946967.1         HQACMSPCPT TKNGVQPFRM ASA-TANCAK MVEYALHNGY D-HVVGMQMG PQTGDAR-TF  525
KFO69021.1             HQACMSPCPT TKRGFQPMRM ASA-TANCAK IIEYVFTSGF D-PAVSMQIG AETPDAA-TF  526
WP_027352796.1         AQACIVPAPE TKHGCMPMRY SSC-TTLGSK CMELALWNGF N-PVFNMQIG PKTGDPC-EM  523
AAS96745.1             NYGCVSTGIC GKGSFITQED QP----CLAK VVELMLNNGK C-PVTKKQVG VESGDPT-TF  509
sp|P09373.2|PFLB_ECOLI IACCVSPMIV GKQ----MQF FGA-RANLAK TMLYAINGGV D-EKLKMQVG PKSEPIKGDV  470

                                 610        620        630        640        650        660
Consensus              KTFEELWEAF RKQYQYAISL LIRTKDVSRY FEGKFLQMPF VSALDDGCME LGM--DANAL  584
ABB31773.1             KTFEQLWEAF RSQYAYATSK VIRAKDIMRY YESKFLQMPF VSSIDDGCME LGI--DSMEL  603
WP_004511527.1         KTFEQLWEAF RSQYAYATSK VIRAKDIMRY YESKFLQMPF VSSIDDGCME LGI--DSMEL  603
CAO72221.1             KTYEELYEAF KVQYQYAVSL VIKCKDTMRY FEGKFLQMPF ASSLDDGCME LGR--DGCEL  603
```

```
CCK78310.1              KTYEELYEAF KVQYQYAVSL VIKCKDTMRY FEGKFLQMPF ASSLDDGCME LGR--DGCEL  603
AIS23708.1              TAFEELWEAF RKQYQYAIAL CIRAKDVSRH FEGKYIQMPF VSAIDDGCME LGM--DANVL  601
AAK50372.1              KTFEDIWEAF RKQYQYAINL GISTKDVSRY FEQRYLQLPF VSAIDDGCME FGM--DACAL  605
BAC05501.1              KTFEDVWEAF REQYQYAINL CICTKDVSRY FEQRFLQMPF VSAIDDGCME LGM--DACAL  605
AAC38454.1              KSFEDVWEAF RKQYQYAINL CISTKDVSRY FEQRFLQMPF VSAIDDGCME LGM--DACAL  605
BAD42366.1              KTFEDLWEAF RKQYQYAIAL CIRAKDVSRY FEGRILQMPF VSAIDDGCME LGV--DANVL  601
CAI07159.1              ATFDQLWEAF RKQYQYAIAL AIRCKDVSRT MECRFLQMPF VSALDDGCME LGM--DANAL  602
WP_011236884.1          ATFDQLWEAF RKQYQYAIAL AIRCKDVSRT MECRFLQMPF VSALDDGCME LGM--DANAL  602
CAA05052.1              ATFDQLWEAF RKQYQYAIAL AIRCKDVSRT MECRFLQMPF VSALDDGCME LGM--DANAL  602
WP_107221981.1          ATFDQLWEAF RKQYQYAIAL AIRCKDVSRT MECRFLQMPF VSALDDGCME LGM--DANAL  602
LMHFDP_300810           KTFDELWNAF RIQNDYVNDM IWRSKDITRK LQMEYLQLPF LSSLDDGCVE LGV--DGTRL  595
WP_155321809.1          KTFDELWEVF RKQSDYVNDM LWRTKDVTRK LQAKYLQLPF LSSLDDGCME RGI--DAVSN  596
WP_155306556.1          QTFEELFEAF KTQFGYCSDL VWRAKDVTRH FQGHYCQLPF VSSLDDGCME KGV--DAMVL  596
WP_014956012.1          KSFDEVWNAV KLQLRYAIEL SLRSKDVGRI MESKYLCCPF ISSIDDGCVE KGM--DANEL  563
CCK78655.1              KSFDEVWNAV KLQLRYAIEL SLRSKDVGRI MESKYLCCPF ISSIDDGCVE KGM--DANEL  563
CAO72219.1              KTFEELFEAF EKQVEFATAL HYRNKDVTRR AEIKFIESPF VASLDDACMD DGV--GAFVD  580
CAO72220.1              KTFEELFEAF EKQVEFATAL HYRNKDVTRR AEIKFIESPF VASLDDACMD DGV--GAFVD  580
CAO72222.1              KTFEELFEAF EKQVEFATAL HYRNKDVTRR AEIKFIESPF VASLDDACMD DGV--GAFVD  580
CAO03074.1              KDFEDLFERW TVQLKWLMNL LVRTVNLGRF KDPEFFGRPF LSAITERAVE HGI--DAVSP  588
ACL03428.1              HDFEDLFQAW VKQMEWLTSL LVRTVNLGRY KDPEFFGRPF LSGMSERSVE SGL--DVVSP  582
WP_012610862.1          HDFEDLFQAW VKQMEWLTSL LVRTVNLGRY KDPEFFGRPF LSGMSERSVE SGL--DVVSP  582
ABH11460.1              HDFEDLFQAW VKQMEWLTSL LVRTVNLGRY KDPEFFGRPF LSGMSERSVE SGL--DVVSP  584
ABH11461.1              TDFEQLFDAW TRQMQWLLSL LVRTVNLGRY KDAEFYGRPL LSGITERAVE RGI--DAVNP  583
WP_015946967.1          TDFEQLFDAW TRQMQWLLSL LVRTVNLGRY KDAEFYGRPL LSGITERAVE RGI--DAVNP  583
KFO69021.1              TSFEQVYAAW ITQMKTIFSI LTRAVNRARI LGAELTPRPF LSAVSERSVE SGL--DVLTP  584
WP_027352796.1          -TFDQLMDAF IEQFKVIHWD AVKIRNIVHH IE-EIHGRPH LSATYEMCVE DGI--NAFTR  579
AAS96745.1              TTFEEVYEAT KKQLDHLFNI SRKHSDLSQM ARLQVVPSVF RSAMYDGCID KGMCEEAGGT  569
sp|P09373.2|PFLB_ECOLI  LNYDEVMERM DHFMDWLAKQ YITALNIIHY MHDKYSYEAS LMALHDRDVI RTM---ACGI  527

                                  670        680        690        700        710        720
Consensus               S-EQPNGWHN PI-TTIVAAN SLVAIKKLVF DDKKYTMEQL LDALKANWEG YEEMRLDFK-  641
ABB31773.1              S-EQPNGWHN PI-TTVVAAN SLVAIKKLIY DEKKYTMAQL VTALRANWHG YEDMRQDFL-  660
WP_004511527.1          S-EQPNGWHN PI-TTVVAAN SLVAIKKLIY DEKKYTMAQL VTALRANWHG YEDMRQDFL-  660
CAO72221.1              S-EQPNGWHN PI-TTIVAAN SMVAIKKLIY DDKKYTMKQL LDALKANWEG YEEMHKDFK-  660
CCK78310.1              S-EQPNGWHN PI-TTIVAAN SMVAIKKLIY DDKKYTMKQL LDALKANWEG YEEMHKDFK-  660
AIS23708.1              S-EQPNGWHN PI-TNIVAAN SLVAIKKLIF DEKRYTMAQL LEALQANWEG YEEMRQDFK-  658
AAK50372.1              S-EQPNAWHN EV-STVVAAN SLVAIKKLVF EEKKYTLEQL SQALKANWEG FEEMRVDFK-  662
BAC05501.1              S-EQPNGWHN PI-TTIVAAN SLVAIKKLVF EEKKYTLEQL SQALKANWEG FEEMRVDFK-  662
AAC38454.1              S-EQPNGWHN PI-TTIVAAN SLVAIKKLVF EEKKYTLEQL SQALKANWEG FEEMRVDFK-  662
BAD42366.1              S-EQPNGWHN PI-TTIVAGN SLVAIKKLIF DDKKYTMEQL VEALKANWEG HEEMRLDFK-  658
CAI07159.1              S-EQPNGWHN PI-TTIVAGN SLVAIKKLIY DEKKYTMEQL MDALKANWEG YEEMRRDFK-  659
WP_011236884.1          S-EQPNGWHN PI-TTIVAGN SLVAIKKLIY DEKKYTMEQL MDALKANWEG YEEMRRDFK-  659
CAA05052.1              S-EQPNGWHN PI-TSIVAGN SLVAIKKLIY DEKKYTMAQL MDALQANWEG YEEMRRDFK-  659
WP_107221981.1          S-EQPNGWHN PI-TSIVAGN SLVAIKKLIY DEKKYTMAQL MDALQANWEG YEEMRRDFK-  659
LMHFDP_300810           A-ELPNPWLQ VH-TAIAACN SMIAIKKLVY EDKKYTMAQL IEALHMNWEG YEEMRLDFL-  652
WP_155321809.1          T-ELPNPWLQ VH-TAIVACN SLTAIKKLVY DEKKYTMAQL IEALHANWEG YEEMRRDFA-  653
WP_155306556.1          S-ELANPWHN CM-TNIVACN SLIACKKLIY DDKKYTMDEL IEALHANWEG YEEMHKDFK-  653
WP_014956012.1          A-EVANPWHN VIGGSVVVID SMAAIKKLVF EDKKYTMAEL MDALRNNWEG KEEMRLDFW-  621
CCK78655.1              A-EVANPWHN VIGGSVVVID SMAAIKKLVF EDKKYTMAEL MDALRNNWEG KEEMRLDFW-  621
CAO72219.1              K-TYPNPWNN TP-GEQTAAD SLAAVKKLVF DDKKYTMEEV VNAMKANFDG YEEMRKDML-  637
CAO72220.1              K-TYPNPWNN TP-GEQTAAD SLAAVKKLVF DDKKYTMEEV VNAMKANFDG YEEMRKDML-  637
CAO72222.1              K-TYPNPWNN TP-GEQTAAD SLAAVKKLVF DDKKYTMEEV VNAMKANFEG HEEMRKDML-  637
CAO03074.1              EGERGNAWVT AF-TWIENVD SMAAIKKLVF DDKKYTMSQL IDALEAEWDG YEQMRLDFVK  647
ACL03428.1              VGDRGNCWVT AF-TWVENID SLAAVKKLVF DDKKYTMEQL LTALKANWDG YEEMRLDFVN  641
WP_012610862.1          VGDRGNCWVT AF-TWVENID SLAAVKKLVF DDKKYTMEQL LTALKANWDG YEEMRLDFVN  641
ABH11460.1              VGDRGNCWVT AF-TWVENID SLAAVKKLVF DDKKYTMEQL LTALKANWDG YEEMRLDFVN  643
ABH11461.1              EGERGNCWIT GF-TWVENAD SLAAVKKLVF DDKKYTMDQL ITALESNWDG YEQMRLDFVN  642
WP_015946967.1          EGERGNCWIT GF-TWVENAD SLAAVKKLVF DDKKYTMDQL ITALESNWDG YEQMRLDFVN  642
KFO69021.1              SISQGNSWIT AF-TWVENAD SLAAIKKLVF EEKKYTMAEL KKALADDWQG HEEMRLDFVK  643
WP_027352796.1          K-EYGNAWVT TF-IWMDGCD ALVACKKLIY DEKKYTMAQL LEFLKANWEG YEKERMDFV-  636
AAS96745.1              RYPQVNPIMT ---AGIDAAN SLLAIRYLVF ETKQVTMEKL LEALKANFEG YEDIRKMCF-  625
sp|P09373.2|PFLB_ECOLI  A--------- ---GLSVAAD SLSAIK---Y AKVKPIRDED GLAIDFEIEG E---------  563

                                  730        740        750        760        770        780
Consensus               NAPKWGNDDD YADXIIKRFY EDIIGGEMRK ITNYSGGPV- --LPVGQAVG LYMEXGSRTG  698
ABB31773.1              NAPKWGNDDE YADTIIKKFY EDIIGGEMAK ITNYSGGPV- --LPVGQAVG LYMEIGSRTG  717
WP_004511527.1          NAPKWGNDDE YADTIIKKFY EDIIGGEMAK ITNYSGGPV- --LPVGQAVG LYMEIGSRTG  717
CAO72221.1              AAPKWGNDNE YADATIKDFY EDIIGGEMGR ITNYSGGPV- --LPVGQAVG LYMEIGSRTG  717
```

```
CCK78310.1             AAPKWGNDNE YADATIKDFY EDIIGGEMGR ITNYSGGPV- --LPVGQAVG LYMEIGSRTG  717
AIS23708.1             NAPKWGNDDS YCDEIVTRFF EDVIGGEMAK ITNYSGGPV- --LPVGQAVG VYMEIGARTG  715
AAK50372.1             RAPKWGNDDE YADSIVSRFY EEVIGGELRK ITNYSGAPV- --LPTGQAVG LYMEVGSRMG  719
BAC05501.1             RAPKWGNDDD YADGIITRFY EEIIGGEMRK ITNYSGGPV- --MPTGQAVG LYMEVGSRTG  719
AAC38454.1             RAPKWGNDDD YADGIITRFY EEIIGGEMRK ITNYSGGPV- --MPTGQAVG LYMEVGSRTG  719
BAD42366.1             NAPKWGNDDA YCDEIIKNFY EDIVGGEMSK ITNYSGGPV- --RPTGQAVG LYMEVGSRTG  715
CAI07159.1             NAPKWGNDDD AADTLISRFY EEILGGEMMK NINYSGGPV- --KPVGQAVG LYMEVGSRTG  716
WP_011236884.1         NAPKWGNDDD AADTLISRFY EEILGGEMMK NINYSGGPV- --KPVGQAVG LYMEVGSRTG  716
CAA05052.1             NAPKWGNDDD DADVLISRFY EEILGGEMMK NINYSGGPV- --KPTGQAVG LYMEVGSRTG  716
WP_107221981.1         NAPKWGNDDD DADVLISRFY EEILGGEMMK NINYSGGPV- --KPTGQAVG LYMEVGSRTG  716
LMHFDP_300810          NAPKWGNDDD YADSLVKAFY EDVLAEKYKR ITTYSGAHP- --LTGSQAVS LYMIIGTFTG  709
WP_155321809.1         AAPKWGNDDD YADAIVKAYW EDILAEKYKR IVTYSGAHP- --LAGSQAVA VYLHIGSITG  710
WP_155306556.1         DAPKWGNDDD YADEIVKRVY EEIFAGKFKE ITNYSGAHP- --MPGGQAVG AYLFIGARTG  710
WP_014956012.1         NAPKFGNDDA YADEIASKYY -DLIADEWKR NTTYSGTYP- --LPLAQSVA GYIVNGPKTA  677
CCK78655.1             NAPKFGNDDA YADEIASKYY -DLIADEWKR NTTYSGTYP- --LPLAQSVA GYIVNGPKTA  677
CAO72219.1             AAPKWGNDDP YVDEIGERVF K-MVADKLME QTTYSGMHP- --LGNPQTVS TFATRAPRIG  693
CAO72220.1             AAPKWGNDDP YVDEIGERVF K-MVADKLME QTTYSGMHP- --LGNPQTVS TFATRAPRIG  693
CAO72222.1             AAPKWGNDDP YVDEIGERIF T-MVADKLME QTTYSGMHP- --LGNPQTVS TFATRAPRIG  693
CAO03074.1             NGPKWGNDDD YVDDIMLRCL SVAA-EHSRN IQCTSGNCW- --PILPENVS GNIHYANIVG  703
ACL03428.1             NAPKWGNDDD YVDDIMLRCL RETA-RHSRV MKCPSGNSW- --PILPENVS GNIHYASIVG  697
WP_012610862.1         NAPKWGNDDD YVDDIMLRCL RETA-RHSRV MKCPSGNSW- --PILPENVS GNIHYASIVG  697
ABH11460.1             NAPKWGNDDD YVDDIMLRCL RETA-RHSRV MKCPSGNSW- --PILPENVS GNIHYASIVG  699
ABH11461.1             KAPKWGNDDD YVDDIMLRCL RTLA-KHSRV MRCTSNNTW- --PISPQNVS GNIHYSSVVG  698
WP_015946967.1         KAPKWGNDDD YVDDIMLRCL RTLA-KHSRV MRCTSNNTW- --PISPQNVS GNIHYSSVVG  698
KFO69021.1             NAPKWGNDDD YVDKIMLRCL HDCA-VFSHE LKDPMGNNW- --PILPENVS GNIHYANVVG  699
WP_027352796.1         KAPKWGNDDP YADDIIVKIH ERVR-DEVCM PCKCWGTHAQ GVPCVPQNVA AYTVCSNLLG  695
AAS96745.1             EAPKHGNDYP EVEHFVQRFY RDVDAIHSAQ GPDCFGYRT- --PLDAYSLS YHNYFGSLMG  682
sp|P09373.2|PFLB_ECOLI -YPQFGNNDP RVDDLAVDLV ERFM-KKIQK LHTYRDAIP- --TQSVLTIT SNVVYGKKTG  618

                                 790        800        810        820        830        840
Consensus              PTPDGRFGGE ALDDGGISPY MGTDKKGPTA VLKSVSKVQK -NQ-KANLLN QRLSVPIM--  754
ABB31773.1             PTPDGRFGGD AGDDGGISPY MGTDKKGPTA VLKSVSKVQK -NQ-KANLLN QRLSVPIM--  773
WP_004511527.1         PTPDGRFGGD AGDDGGISPY MGTDKKGPTA VLKSVSKVQK -NQ-KANLLN QRLSVPIM--  773
CAO72221.1             PTPDGRLGGE AADDGGISPY MGTDHKGPTA VLKSVSKVQK -NQ-KANLLN QRLSVPIM--  773
CCK78310.1             PTPDGRLGGE AADDGGISPY MGTDHKGPTA VLKSVSKVQK -NQ-KANLLN QRLSVPIM--  773
AIS23708.1             PTPDGRFGGE AADDGGISPY MGTDKKGPTA VLRSVSKVQK -NQ-KANLLN QRLSVPIM--  771
AAK50372.1             PTPDGRFGGE AADDGGISPY MGTDKKGPTA VLRSVSKVQK -NQ-KANLLN QRLSVPIM--  775
BAC05501.1             PTPDGRFGGE AADDGGISPY MGTDKKGPTA VLRSVSKVQK -NQ-KGNLLN QRLSVPIM--  775
AAC38454.1             PTPDGRFGGE AADDGGISPY MGTDKKGPTA VLRSVSKVQK -NQ-KGNLLN QRLSVPIM--  775
BAD42366.1             PTPDGRLGGE AADDGGISPY MGTDKKGPTA VLRSVSKVQK -NQ-KANLLN QRLSVPIM--  771
CAI07159.1             PTPDGRFGGE AADDGGISPY SGTDKKGPTA VLRSVSKVQK -NQ-KANLLN QRLSVPIM--  772
WP_011236884.1         PTPDGRFGGE AADDGGISPY SGTDKKGPTA VLRSVSKVQK -NQ-KANLLN QRLSVPIM--  772
CAA05052.1             PTPDGRFGGE AADDGGISPY SGTDKKGPTA VLRSVSKVQK -NQ-KANLLN QRLSVPIM--  772
WP_107221981.1         PTPDGRFGGE AADDGGISPY SGTDKKGPTA VLRSVSKVQK -NQ-KANLLN QRLSVPIM--  772
LMHFDP_300810          PTPDGRFGGE ALDDGGISPM AGTDKNGPTA VLRSMSKLDS -SKFKFNLLN QRLSVPLM--  766
WP_155321809.1         PTPDGRFGGE ACDDGGVSPM AGTDKLGPTA VLRSLSKIDS -SKFKFNLLN QRLSVPLM--  767
WP_155306556.1         PTPDGRYGGE ACDDGGISPM AGTDTKGPTA VIKSVSKVDS -ETQKFNLLN QRLSVPLM--  767
WP_014956012.1         ATANGRHAGE ALDDGGCSPY MGCDKSGPTA VLKSVSKIDA -SKHKGILLN QRLSTVLM--  734
CCK78655.1             ATANGRHAGE ALDDGGCSPY MGCDKSGPTA VLKSVSKIDA -SKHKGILLN QRLSTVLM--  734
CAO72219.1             ALPFGKLHGE VLHDGGSSPY VGLDKKGPTA VLKSVAHIPY -DRYKGVQFN QRLPVSIM--  750
CAO72220.1             ALPFGKLHGE VLHDGGSSPY VGLDKKGPTA VLKSVAHIPY -DRYKGVQFN QRLPVSIM--  750
CAO72222.1             ALPFGKLHGE VLHDGGSSPY VGLDKKGPTA VLKSVAHIPY -DRYKGVQFN QRLPVSVM--  750
CAO03074.1             ALPNGRRRGD ALYDGGVSPG PGLDKAGPTA VLKSVGKIDH VNQGRSFLLN QRLSPTQL--  761
ACL03428.1             ALPNGRRCGD ALYDGGISPG PGLDKKGPSA VLKSCGKIDH VSDGRAFLLN QRLSPTQL--  755
WP_012610862.1         ALPNGRRCGD ALYDGGISPG PGLDKKGPSA VLKSCGKIDH VSDGRAFLLN QRLSPTQL--  755
ABH11460.1             ALPNGRRCGD ALYDGGISPG PGLDKKGPSA VLKSCGKIDH VSDGRAFLLN QRLSPTQL--  757
ABH11461.1             ALPNGRRLGD ALYDGGISPG PGLDKKGPTA VLKSCGKIDH VGDGRAFLLN QRLSPTQM--  756
WP_015946967.1         ALPNGRRLGD ALYDGGISPG PGLDKKGPTA VLKSCGKIDH VGDGRAFLLN QRLSPTQM--  756
KFO69021.1             ALPSGRRRGD ALYDGGISPG PGLDKKGPTA VLKSCGKIDH ITDGRAFLLN QRLSPTQL--  757
WP_027352796.1         ALPNGRRLGD TCYDGGCSPG AGNDKKGPTA VLNSVGKLEH ENMFRANLLN QRLSPTQL--  753
AAS96745.1             ALPNGRKAGV ALTDGSVSAM PGTDHEGITA LIKSGAEAID TVRYGANHFN VKFNPSVI--  740
sp|P09373.2|PFLB_ECOLI NTPDGRRAG- APFGPGANPM HGRDQKGAVA SLTSVAKLPF AYAKDGISYT FSIVPNALGK  677

                                 850        860        870        880        890        900
Consensus              ----RSKHGF DIWHAYMKTW HDL-NIDHVQ FNVVSTEEMR AAQKEPEKHQ DLIVRVAGYS  809
ABB31773.1             ----RSVHGF TIWKSYMDAW EKL-NIDHVQ FNCVSTAEMK AAQKEPEKHQ DLIVRVSGFS  828
WP_004511527.1         ----RSVHGF TIWKSYMDAW EKL-NIDHVQ FNCVSTAEMK AAQKEPEKHQ DLIVRVSGFS  828
CAO72221.1             ----RSKHGF DIWNAYMKTW HDL-KIDHIQ FNVVSTAEMK AAQKEPEKHQ DLIVRVSGFS  828
```

```
CCK78310.1                 ----RSKHGF DIWNAYMKTW HDL-KIDHIQ FNVVSTAEMK AAQKEPEKHQ DLIVRVSGFS  828
AIS23708.1                 ----RSTHGF NIWKAYMDTW HDL-NIDHVQ FNVLSTEEMR AAQREPEKHQ DLIVRVSGYS  826
AAK50372.1                 ----RSKHGF EIWNAYMKTW HEL-NIDHVQ FNVVSTDEMR AAQREPEKHS DLIVRVSGYS  830
BAC05501.1                 ----RSKHGF EIWNSYMKTW HDL-NIDHVQ FNVVSTDEMR AAQREPEKHH DLIVRVSGYS  830
AAC38454.1                 ----RSKHGF EIWNSYMKTW HDL-NIDHVQ FNVVSTDEMR AAQREPEKHH DLIVRVSGYS  830
BAD42366.1                 ----RSAHGF DIWHAYMNTW HDL-NIDHVQ FNVVSTEEMR AAQREPEKHH DLIVRVSGYS  826
CAI07159.1                 ----RSKHGF DIWHAYMDTW HEL-NIDHVQ FNVVSTEEMK AAQREPEKHQ DLIVRVSGFS  827
WP_011236884.1             ----RSKHGF DIWHAYMDTW HEL-NIDHVQ FNVVSTEEMK AAQREPEKHQ DLIVRVSGFS  827
CAA05052.1                 ----RSKHGF DIWHAYMDTW HDL-NIDHVQ FNVVSTEEMK AAQREPEKHQ DLIVRVSGFS  827
WP_107221981.1             ----RSKHGF DIWHAYMDTW HDL-NIDHVQ FNVVSTEEMK AAQREPEKHQ DLIVRVSGFS  827
LMHFDP_300810              ----RSKHGF DIWHAYMKTW HDM-KIDHVQ FNCVTTEEMK AAQVEPEQHE DLIVRVAGYS  821
WP_155321809.1             ----RSKHGF DIWHAYMKTW HDL-KIDHVQ FNCVSTEEML AAQKEPEQHE DLIVRVAGYS  822
WP_155306556.1             ----RSTHGF DIWHAYMKTW CDL-KCDHVQ FNCVSTEEMR AAQKEPEKHG DLIVRVAGFS  822
WP_014956012.1             ----NSDAGF DLWHAYMKTW HSL-GIDHVQ FNVISQEDMK AAQIEPEKYT DTLVRIAGYS  789
CCK78655.1                 ----NSDAGF DLWHAYMKTW HSL-GIDHVQ FNVISQEDMK AAQIEPEKYT DTLVRIAGYS  789
CAO72219.1                 ----RGDKGF QVWSAYMKAW HDL-NIDHVQ FNVVETKDML EAQKEPEKWE SLIVRIAGYS  805
CAO72220.1                 ----RGDKGF QVWSAYMKAW HDL-NIDHVQ FNVVETKDML EAQKEPEKWE SLIVRIAGYS  805
CAO72222.1                 ----RGDKGF QVWTAYMKAW HDL-NIDHVQ FNVVDTKDML EAQKEPEKWQ SMIVRIAGYS  805
CAO03074.1                 ----AGDKGF QLWNSYVRTW AEL-GIDHIQ FNVISDKVLR AAQNDPEGYQ EVIVRVAGYS  816
ACL03428.1                 ----AGEKGY QLWKAYIRTW ADL-GLDHVQ FNMVSDETLR AAQKDPEKYS EVIVRVAGYS  810
WP_012610862.1             ----AGEKGY QLWKAYIRTW ADL-GLDHVQ FNMVSDETLR AAQKDPEKYS EVIVRVAGYS  810
ABH11460.1                 ----AGEKGY QLWKAYIRTW ADL-GLDHVQ FNMVSDETLR AAQKDPEKYS EVIVRVAGYS  812
ABH11461.1                 ----AGEKGY QLWRAYMRTW ADL-GLDHIQ FNMVSDKTLR AAQKDPEKYQ EVIVRVAGYS  811
WP_015946967.1             ----AGEKGY QLWRAYMRTW ADL-GLDHIQ FNMVSDKTLR AAQKDPEKYQ EVIVRVAGYS  811
KFO69021.1                 ----AGEKGY SFWKSYMDTW YNL-GLDHIQ FNCVSDETLR SAQREPEKYQ EVIVRVAGYS  812
WP_027352796.1             ----AGDKGF DIWNSYIQSW CDL-GINHVQ FNIVDNETLL AAQEKPEDFE EMIVRVAGYS  808
AAS96745.1                 ----EGPAGA RTLVSLIKTY CDF-GGSHIQ FNCVSSDTLK DAQCKPQEYA DLVVRVAGFS  795
sp|P09373.2|PFLB_ECOLI DDEVRKTNLA GLMDGYFHHE ASIEGGQHLN VNVMNREMLL DAMENPEKYP QLTIRVSGYA  737

                                 910        920        930
Consensus                  ARFVDIPTYG QNTIIARTEQ XFGAQDLEFL NVELXDAK                     847
ABB31773.1                 ARFVDIPTYG QNTIIARNEQ AFGADDLEYL NTQL----                     862
WP_004511527.1             ARFVDIPTYG QNTIIARNEQ AFGADDLEYL NTQ-----                     861
CAO72221.1                 SRFVDIPTYG QNTIIARNEQ QFGAEDFEYL NLDI----                     862
CCK78310.1                 SRFVDIPTYG QNTIIARNEQ QFGAEDFEYL NLDI----                     862
AIS23708.1                 ARFVDLPTFG QNTIIARHEQ EFSGEDMEFL NVDL----                     860
AAK50372.1                 ARFVDLPTYG QNTIIARQEQ DFSASDLEFL NVEI----                     864
BAC05501.1                 ARFVDIPTYG QNTIIARQEQ DFSASDLEFL NVEI----                     864
AAC38454.1                 ARFVDIPTYG QNTIIARQEQ DFSASDLEFL NVEI----                     864
BAD42366.1                 ARFVDIPTYG QNTIIARHEQ DFSANDLEFL NCDL----                     860
CAI07159.1                 ARFVDIPTYG QNTIIARNEQ DFNAQDLEFL NAEL----                     861
WP_011236884.1             ARFVDIPTYG QNTIIARNEQ DFNAQDLEFL NAEL----                     861
CAA05052.1                 ARFVDIPTYG QNTIIARNEQ NFNAQDLEFL NVEL----                     861
WP_107221981.1             ARFVDIPTYG QNTIIARNEQ NFNAQDLEFL NVEL----                     861
LMHFDP_300810              AKFVDVPTYG QNTIIARTEQ KFGPNQFDDL EVEFTDAK                     859
WP_155321809.1             ARFVDISTYA QNTIIKRNVQ GFGPAEFDKF SVEFSEA-                     859
WP_155306556.1             ARFVDVSTYG QNTIIARNEQ TFGPYDFDEL GVEFADN-                     859
WP_014956012.1             AKFIDLARYS QDTIIARTEQ DMAG------ --------                     813
CCK78655.1                 AKFIDLARYS QDTIIARTEQ DMAG------ --------                     813
CAO72219.1                 ARFVSLPKNA QDAIIARNEQ QIG------- --------                     828
CAO72220.1                 ARFVSLPKNA QDAIIARNEQ QIG------- --------                     828
CAO72222.1                 ARFVSLPRNA QDSIIARTEQ PVG------- --------                     828
CAO03074.1                 AHFIDISRKT QDNIIQRTVQ GLG------- --------                     839
ACL03428.1                 AHFVDISRKT QDNIIQRTVQ GI-------- --------                     832
WP_012610862.1             AHFVDISRKT QDNIIQRTVQ GI-------- --------                     832
ABH11460.1                 AHFVDISRKT QDNIIQRTVQ GI-------- --------                     834
ABH11461.1                 AHFVDISRKT QDNIIQRTVQ GI-------- --------                     833
WP_015946967.1             AHFVDISRKT QDNIIQRTVQ GI-------- --------                     833
KFO69021.1                 AHFVDISRKT QDNIIQRTVQ GIG------- --------                     835
WP_027352796.1             AQFVGLNKKT QDTIIARTIQ EL-------- --------                     830
AAS96745.1                 AYFTRLDKGV QNEIIKRTEY KN-------- --------                     817
sp|P09373.2|PFLB_ECOLI VRFNSLTKEQ QQDVITRTFT QSM------- --------                     760
```
